# High-Throughput Genetic Screens Identify a Large and Diverse Collection of New Sporulation Genes in *Bacillus subtilis*

**DOI:** 10.1371/journal.pbio.1002341

**Published:** 2016-01-06

**Authors:** Alexander J. Meeske, Christopher D. A. Rodrigues, Jacqueline Brady, Hoong Chuin Lim, Thomas G. Bernhardt, David Z. Rudner

**Affiliations:** Department of Microbiology and Immunobiology, Harvard Medical School, Boston, Massachusetts, United States of America; Indiana University, UNITED STATES

## Abstract

The differentiation of the bacterium *Bacillus subtilis* into a dormant spore is among the most well-characterized developmental pathways in biology. Classical genetic screens performed over the past half century identified scores of factors involved in every step of this morphological process. More recently, transcriptional profiling uncovered additional sporulation-induced genes required for successful spore development. Here, we used transposon-sequencing (Tn-seq) to assess whether there were any sporulation genes left to be discovered. Our screen identified 133 out of the 148 genes with known sporulation defects. Surprisingly, we discovered 24 additional genes that had not been previously implicated in spore formation. To investigate their functions, we used fluorescence microscopy to survey early, middle, and late stages of differentiation of null mutants from the *B*. *subtilis* ordered knockout collection. This analysis identified mutants that are delayed in the initiation of sporulation, defective in membrane remodeling, and impaired in spore maturation. Several mutants had novel sporulation phenotypes. We performed in-depth characterization of two new factors that participate in cell–cell signaling pathways during sporulation. One (SpoIIT) functions in the activation of σ^E^ in the mother cell; the other (SpoIIIL) is required for σ^G^ activity in the forespore. Our analysis also revealed that as many as 36 sporulation-induced genes with no previously reported mutant phenotypes are required for timely spore maturation. Finally, we discovered a large set of transposon insertions that trigger premature initiation of sporulation. Our results highlight the power of Tn-seq for the discovery of new genes and novel pathways in sporulation and, combined with the recently completed null mutant collection, open the door for similar screens in other, less well-characterized processes.

## Abstract 2

Transposon sequencing enables the recovery of virtually all previously characterized genes required for the differentiation of the bacterium *Bacillus subtilis* into a dormant spore and identifies 24 new ones.

## Author Summary

When starved of nutrients, the bacterium *Bacillus subtilis* differentiates into a dormant spore that is impervious to environmental insults. Decades of research have uncovered over 100 genes required for spore formation. Molecular dissection of these genes has revealed factors that act at every stage of this developmental process. In this study, we used a high-throughput genetic screening method called transposon sequencing to assess whether there were any sporulation genes left to be discovered. This approach identified virtually all of the known sporulation genes, as well as 24 new ones. Furthermore, transposon sequencing enabled the discovery of two new sets of mutants in which the sporulation process was either delayed or accelerated. Using fluorescence microscopy, we determined the developmental stage at which each mutant was impaired and discovered mutants that are delayed in initiation of sporulation, or defective in morphogenesis, cell–cell signaling, or spore maturation. Our findings exemplify the utility of transposon sequencing to uncover new biology in well-studied processes, suggesting that it could similarly be used to identify novel genes required for other aspects of bacterial physiology, such as natural competence, stationary phase survival, or the responses to cell envelope stress and DNA damage.

## Introduction

The morphological process of spore formation in *Bacillus subtilis* has been studied for over 50 years and constitutes one of the most well-characterized developmental pathways in biology [1–4]. Its molecular dissection has contributed to our understanding of diverse biological processes, including cell fate determination, signal transduction, membrane and cell wall remodelling, subcellular protein localization, and chromosome dynamics [5–8].

Underlying this seemingly simple process is a set of highly orchestrated morphological events that are both driven by and coupled to developmental programs of gene expression. Upon nutrient limitation and in response to population density, *B*. *subtilis* enters the sporulation pathway. The first landmark event in this process is an asymmetric division, generating a large cell (called the mother cell) and a smaller cell (the prospective spore or forespore). Shortly after polar division, the mother cell membranes migrate around the forespore in a phagocytic-like process called engulfment, generating a cell within a cell. Membrane fission at the cell pole releases the forespore into the mother cell cytoplasm. The mother then assembles a set of protective layers around the forespore, while the spore prepares for dormancy. When the spore is fully mature, the mother cell lyses, releasing it into the environment.

This differentiation process takes 5–7 h to complete and is controlled by a series of stage- and compartment-specific transcription factors. Although the mother and forespore follow distinct developmental programs of gene expression, they remain linked to each other through cell–cell signalling pathways. Entry into sporulation is governed by two transcriptional regulators: the response regulator Spo0A and the stationary phase sigma factor σ^H^. The proteins produced under their control prevent new rounds of DNA replication, remodel the replicated chromosomes, and shift the cell division site from a medial to a polar position. These regulators are also responsible for the expression of compartment-specific transcription factors that are held inactive until polar division is complete. Asymmetric division triggers the activation of the first forespore-specific sigma factor σ^F^. σ^F^ controls the expression of a collection of forespore proteins, including a secreted signalling molecule that activates the first mother cell transcription factor σ^E^. The proteins produced under σ^E^ control prevent a second division at the opposite cell pole and drive the migration of the mother cell membranes around the forespore. During this process, a transenvelope complex is assembled in the membranes that separate the mother cell and forespore. This complex, which resembles a specialized secretion system, allows the mother to maintain forespore physiology and developmental gene expression under the control of the late-acting transcription factor σ^G^. After the completion of engulfment, σ^G^ produces two signalling proteases that are secreted into the space between the mother and forespore membranes, where they trigger the activation of the mother cell transcription factor σ^K^. A large set of proteins produced under the control of σ^E^ and σ^K^ in the mother cell are responsible for assembling a thick layer of peptidoglycan between mother cell and forespore membranes and a multi-layered proteinaceous coat on the spore exterior. σ^G^ in the forespore controls the expression of proteins that prepare the spore for dormancy, including DNA binding proteins that compact and protect the spore chromosome and a membrane complex that imports dipicolinic acid, a compound that facilitates spore dehydration. Finally, a set of cell wall degrading enzymes produced in the mother cell under σ^K^ control trigger mother cell lysis and release of the dormant spore.

Five decades of research have led to the identification and characterization of over 100 genes that are required for successful completion of this developmental process. A pigmented protein produced in the mother cell and assembled around the developing spore gives rise to opaque beige colonies on sporulation agar plates [9]. Many sporulation mutants were originally identified in classical genetic screens based on their failure to produce this pigment [10–12]. Some of these genes are absolutely required for spore formation, while most play important but nonessential roles with mutant sporulation phenotypes ranging over eight orders of magnitude [13]. Advances in the identification and characterization of sporulation genes have been punctuated by the introduction of new technologies. These include the use of the Tn917 transposon (and *lacZ*-containing variants) to map sporulation mutants and identify sporulation-induced genes [14,15], cytological approaches to localize sporulation proteins and monitor spore morphogenesis [16–18], and transcriptional profiling to define entire sporulation-specific regulons [19–25]. More recently, taking advantage of the groundswell of sequenced bacterial genomes, novel sporulation genes have been unearthed through phylogenetic profiling [26,27].

Here, we screened for new sporulation genes using a transposon-sequencing (Tn-seq) approach [28]. Our screen identified virtually all of the known sporulation genes and 24 new ones that we have characterized by taking advantage of the ordered *B*. *subtilis* null mutant collection. These new genes play roles in several stages of development, including initiation, morphogenesis, cell–cell signalling, and spore maturation. Furthermore, by repeating the Tn-seq screen with cells undergoing sporulation for 5 h instead of 24, we discovered that a large set of uncharacterized sporulation-induced genes are required for timely development. Finally, this second screen also identified a novel set of transposon insertions that induce premature initiation of sporulation, giving the appearance of accelerated spore maturation. Collectively, this approach identified new sporulation genes, novel phenotypes, and new pathways, and underscores the utility of Tn-seq to uncover new biology in well-studied processes.

## Results

### A Screen for Mutants Defective in Sporulation

**Fig 1:**
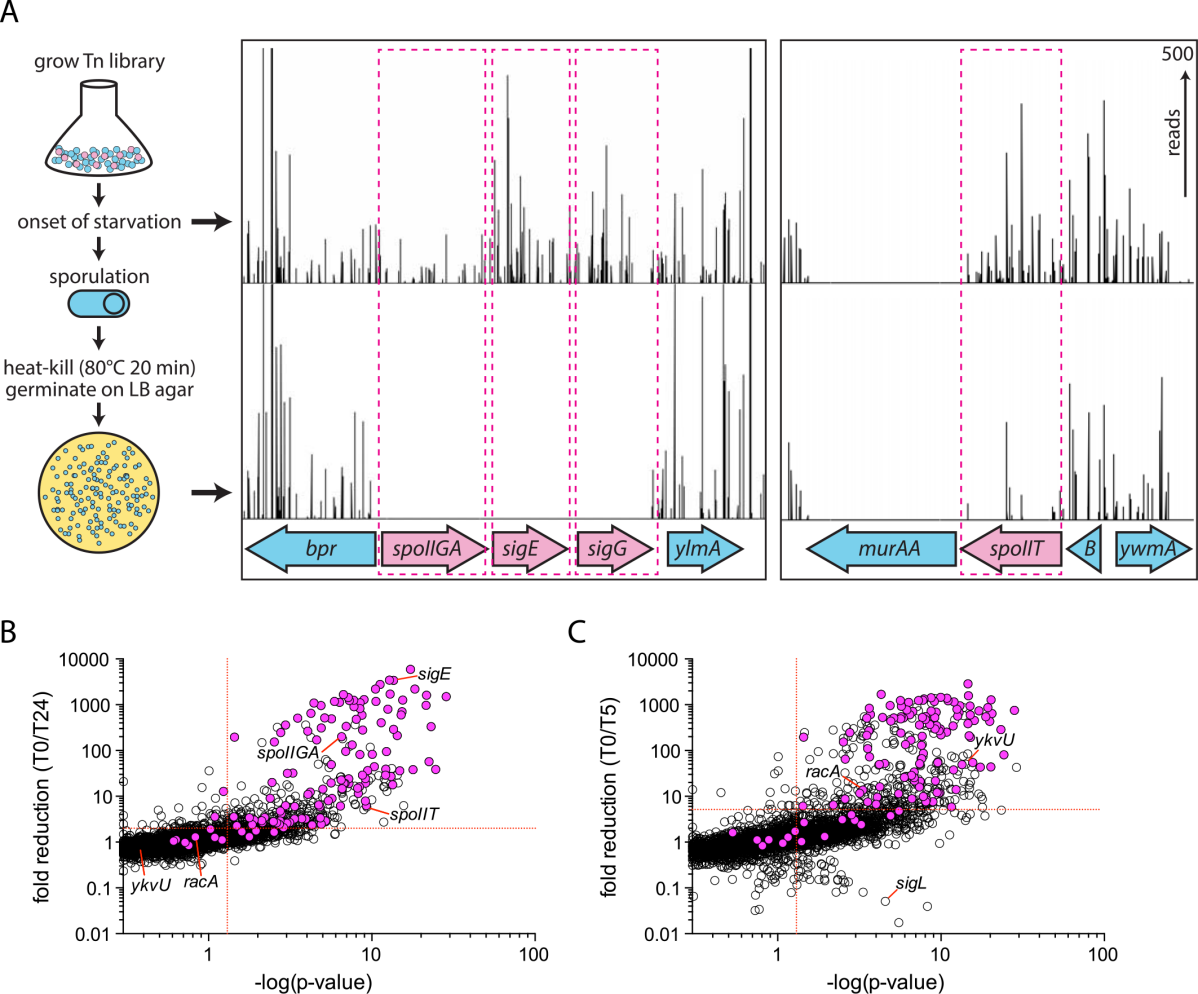
Tn-seq screen for new sporulation genes. **(A)** Flow diagram of the screen. A Mariner-based transposon library was grown in sporulation medium. At the onset of starvation (T0), a sample was removed. The remaining culture was allowed to sporulate for 24 h (T24). Heat treatment at 80°C for 20 min killed vegetative cells and sporulation-defective mutants (pink circles). The surviving spores (blue circles) were germinated on LB agar plates and the resulting colonies pooled. The transposon insertion sites and their abundance were determined for the T0 sample and the T24 heat-resistant colony forming units (CFU) by deep sequencing and mapping onto a reference genome (see Methods). Transposon insertion profiles from two regions of the genome are depicted. The height of each vertical line reflects the number of sequencing reads at this position. Transposon insertions in previously identified sporulation genes such as *spoIIGA*, *sigE*, and *sigG* were statistically underrepresented after sporulation. A new sporulation gene, *spoIIT* (formerly *ywmB*) had 5.8-fold fewer transposon insertions after sporulation. Insertions in the essential *murAA* gene were not tolerated under either condition. **(B)** Scatterplot showing the extent of decrease in transposon insertions in all genes at T24. Previously identified sporulation genes are highlighted in pink. For each gene, the–log of the Mann Whitney *U p*-value comparing the T0 and T24 libraries is plotted against the fold reduction in transposon insertions at T24. The statistical significance thresholds (*p* <0.05, fold reduction >2) are highlighted by dashed red lines. All sporulation genes, including those outside the statistical significance thresholds, are listed in S1 Table. **(C)** Scatterplot showing the extent of decrease in transposon insertions in all genes at T5. The data are displayed as in (B). A more stringent significance threshold for fold reduction (>5-fold) was used for this dataset. The complete list of hits can be found in S2 Table. The raw underlying numerical data for Fig 1B and 1C can be found in S1 Data.

To investigate whether there were additional genes required for sporulation, we performed a high-throughput screen using transposon-sequencing (Tn-seq). We generated a Mariner transposon library containing over 138,000 unique insertions (in 63% of all possible TA dinucleotides) in the *B*. *subtilis* 168 genome (see Methods). On average, each gene was disrupted at 26 unique sites. The library was grown in sporulation medium and a sample was removed at the onset of starvation (T0). The remaining culture was allowed to exhaust its nutrients and sporulate over the next 24 h (T24). The culture was then incubated at 80°C for 20 min to kill vegetative and sporulation-defective cells and the spores were plated on lysogeny broth (LB) agar plates. More than 750,000 colonies from germination-proficient spores were pooled and the transposon insertions at T0 and T24 were mapped by massively parallel DNA sequencing (see Methods). The location and number of reads for each transposon insertion were compared at the onset of starvation and after heat-kill, germination, and outgrowth (for simplicity, referred to as sporulation) (Fig 1 and S1 Fig). We set an initial statistical significance cutoff value of *p* < 0.05 with a 2-fold reduction in transposon insertions at T24 compared to T0 (Fig 1B and S1 Data). More than 350 genes met these initial criteria. Visual inspection of the transposon insertion profiles revealed that ~20% of these genes had very few insertions at T0. An additional ~20% had a modest (2–3-fold) reduction in transposon insertions at T24, but insertions were present throughout the open reading frame. Most of these genes were not considered further (Fig 1 and S1 Fig).

Of the 111 well-characterized sporulation genes, our screen identified 98 (Fig 1 and S1 Fig and S1 Table). Among these were all the genes with strong sporulation mutant phenotypes, including *sigE*, *spoIIGA*, and *sigG* (Fig 1), *spoIIR*, *spoIIIC*, and *spoVB* (S1 Fig). Moreover, we also identified genes like *kinA*, *spoIIB*, and *bofA* for which the sporulation efficiency of the mutants ranges from 1%–20% (S1 Fig and S1 Table) [29–31]. The 13 genes that were not identified in our screen had very mild or no sporulation/germination phenotypes under our assay conditions (S1 Table) (see below). An additional 37 less well-characterized genes have been reported to be required for efficient sporulation, many of which were originally identified by transcriptional profiling [19–24,26,27]. Thirty-five of these genes were identified in our Tn-seq screen (Table 1). Thus, out of the 148 previously identified genes required for sporulation, our screen identified 133 of them. The validation and characterization of 24 additional genes that had not been implicated in spore formation will be described below.

**Table 1:** Independently validated sporulation genes identified by Tn-seq.

section	gene	*p*-value^a^	fold change^b^	description	spo (%)^c^	cytological phenotype
***Newly identified sporulation genes***	*** ***	*** ***	*** ***	*** ***	*** ***	*** ***
	**araR**	1.24 × 10^−12^	24.2	repressor of arabinose utilization	28	no observable phenotype
	**defB**	2.53 × 10^−7^	14	N-formylcysteine deformylase	9	engulfment and fission defects/reduced σG activity
	**gapB**	1.02 × 10^−12^	16.1	GAP dehydrogenase	0.3	delayed entry/thin cells/fewer sporulating cells
	**gidA**	1.87 × 10^−9^	13.8	tRNA-modifying enzyme	23.9	longer cells/some aberrant septa and forespores—similar to Δ*trmE*
	**gtaB**	4.96 × 10^−8^	123.8	UDP-glucose pyrophosphorylase	0.1	smaller and wider cells with small forespores—identical to Δ*pgcA*
	**iolR**	7.01 × 10^−5^	3.2	repressor of myo-inositol catabolism	38	no observable phenotype
	**miaA**	1.27 × 10^−6^	2.7	tRNA-modifying enzyme	16	ND
	**minJ**	2 × 10^−5^	3.5	division site selection	36	delayed entry/aberrant forespores
	**ndk**	7.45 × 10^−5^	4.6	nucleotide diphosphate kinase	13.4	ND
	**nrnA**	3.04 × 10^−6^	6.7	nanoRNase	5	delayed entry/some small forespores with reduced σG activity
	**pgcA**	5.17 × 10^−15^	28.9	UDP-glucose synthesis	0.1	smaller and wider cells with small forespores—identical to Δ*gtaB*
	**rasP**	3.75 × 10^−7^	26.3	intramembrane protease	1.4	delayed entry/thin and oblong forespores
	**resA**	6.56 × 10^−4^	4.9	heme translocase	45.6	delayed entry
	**rex (ydiH)**	3.42 × 10^−7^	26.7	transcriptional repressor	9.2	no observable phenotype
	**smpB**	1.2 × 10^−3^	9.8	tmRNA-binding protein	23	delayed entry
	**speE**	5.16 × 10^−6^	3.7	spermidine synthase	6	delayed entry
	**spoIIT (ywmB)**	4.76 × 10^−10^	5.8	unknown	38	abortive disporic/impaired σE activation
	**spoIIIL (yqzE)**	2 × 10^−3^	15.9	unknown	14	small spores/reduced σG activity
	**trmE**	3.48 × 10^−7^	13.7	tRNA-modifying enzyme	21.9	longer cells/some aberrant septa and forespores—similar to Δ*gidA*
	**ubiE (menH)**	2.41 × 10^−7^	180.8	menaquinone synthesis	12.7	no observable phenotype
	**yaaD (pdxT)**	8.73 × 10^−5^	11	pyridoxal 5′-phosphate synthesis	1.2	ND
	**yerC**	1.15 × 10^−4^	359	putative DNA-binding protein	10	no observable phenotype
	**yqhT**	2.78 × 10^−10^	22.1	Xaa-Pro peptidase	1.2	delayed entry
	**ytxG**	5.8 × 10^−5^	193	general stress protein/sigB/sigH	39.6	aberrant membrane morphologies
***Previously identified but less well characterized sporulation genes***	** **					
	**asnO**	1.58 × 10^−15^	24.2	asparagine synthase (σE regulon)	0	no observable phenotype
	**clpC**	7.79 × 10^−16^	600.7	ATPase subunit of ClpCP	0.4	delayed entry/engulfment defect/small spores/reduced σG activity
	**cotE**	3.23 × 10^−2^	2.3	coat protein (σE and σK regulons)	49.1	ND
	**dacB**	6.54 × 10^−13^	39.3	DD-carboxypeptidase	19.3	no observable phenotype
	**dgkA**	2.56 × 10^−5^	170.3	undecaprenol kinase/cortex synthesis	33.7	ND
	**divIVA**	3.21 × 10^−3^	2.8	division site selection/σF activity	11.1	ND
	**ecsA**	3.51 × 10^−9^	81.5	ABC transporter family	0.1	ND
	**ecsB**	6.79 × 10^−10^	19.5	ABC transporter family	13.4	ND
	**efp**	3 × 10^−7^	200	elongation factor P	1	ND
	**ftsH**	2.14 × 10^−9^	139.1	membrane protease	0	ND
	**gerM**	2 × 10^−7^	96.3	cortex synthesis and/or germination (σE regulon)	0.3	ND
	**kbaA**	3.73 × 10^−2^	3.5	regulator of KinB	74	ND
	**lgt (gerF)**	1.68 × 10^−8^	905.7	lipomodification/germination	7.7	ND
	**mntR**	2.16 × 10^−3^	11.2	trxn regulator of Mn++ transport	8.9	ND
	**prpC**	4.24 × 10^−4^	6	phosphatase	50.7	ND
	**putB (ycgM)**	6.2 × 10^−3^	3.4	proline dehydrogenase (spo0A and σE regulons)	0	no observable phenotype
	**rsfA**	4.12 × 10^−3^	9.1	regulator of σF controlled genes	11.6	ND
	**skfE**	1.42 × 10^−7^	19.1	export of sporulation killing factor	54	ND
	**skfF**	4.1 × 10^−23^	57.3	export of sporulation killing factor	62.3	ND
	**spoVIE (pdaB)**	7.23 × 10^−6^	518	polysaccharide deacetylase (σE regulon)	5	ND
	**spoVIGA (ytrH)**	6.39 × 10^−5^	12.4	cortex synthesis (σE regulon)	17.2	ND
	**spoVIGB (ytrI)**	1.58 × 10^−5^	5.9	spore coat/cortex synthesis	11.9	ND
	**uppP**	2.66 × 10^−8^	132.7	minor UPP phosphatase	0.4	distended intermembrane space
	**yabQ**	2.97 × 10^−11^	2206	cortex synthesis (σE regulon)	0.1	ND
	**ydcC**	3.9 × 10^−6^	8	slightly reduced σG activity (σE regulon)	13.7	small forespores some with reduced σG activity
	**ylbJ**	2.92 × 10^−13^	801	cortex synthesis (σE regulon)	0	no observable phenotype
	**ymdB**	2.64 × 10^−7^	14.7	phosphodiesterase/bistable gene expression	11.4	ND
	**yqfC**	5.22 × 10^−4^	32.3	unknown (σE regulon)	72.9	ND
	**yqfD**	1.54 × 10^−13^	3448	similar to UDP-glucose 4-epimerase (σE regulon)	0	some small spores with reduced σG activity
	**ytvI**	6.68 × 10^−9^	15.1	autoinducer-2 exporter family (σE regulon)	14	no observable phenotype

^**a**^*p*-value based on Mann Whitney *U* test comparing T0 and T24 libraries

^**b**^fold change refers to number of transposon insertions at T0 compared to T24

^**c**^sporulation efficiency (spo) was determine 24 h after starvation and refers to heat-resistant colony forming units (CFU) of null mutant compared to wild-type

^**d**^ND, not determined.

### Tn-seq Identifies Mutants That Delay Sporulation

**Fig 2:**
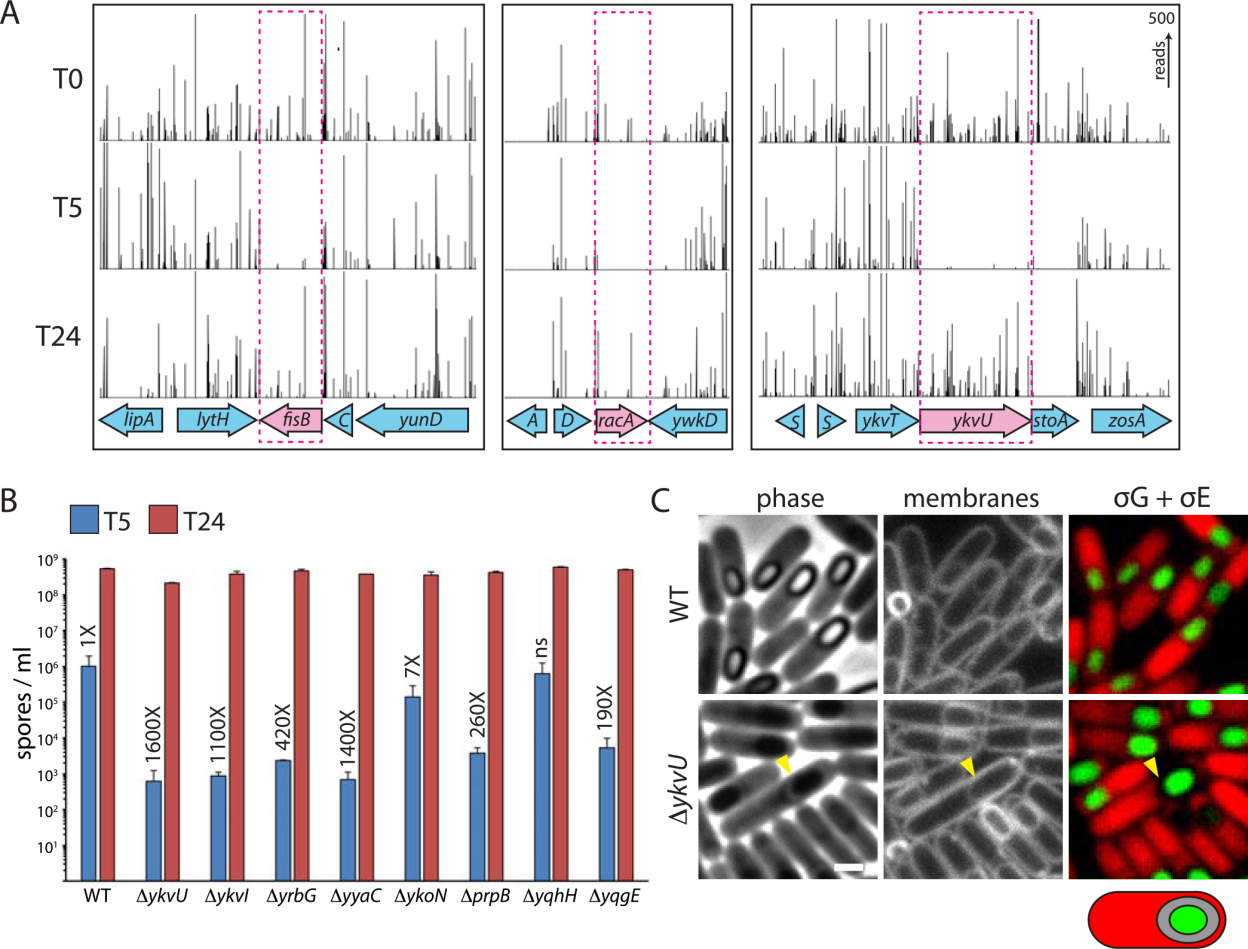
Tn-seq identifies developmentally delayed mutants. **(A)** Transposon insertion profiles from three genomic regions are depicted from libraries prepared from the onset of starvation (T0), 5 h after entry into sporulation (T5), and after 24 h of sporulation (T24). At T24, transposon insertions in two known sporulation genes (*fisB* and *racA*) and a sporulation-induced gene (*ykvU*) are readily detected. However, insertions in these genes are significantly underrepresented at T5. **(B)** Bar graphs of spore titers at hour 5 (in blue) and 24 (in red) of wild-type and indicated null mutants identified by Tn-seq. All eight mutants from the knockout collection had their antibiotic cassette excised. Error bars represent standard error of the mean from two biological replicates. The fold reduction compared to wild type at T5 is indicated above each bar. The raw underlying numerical data for Fig 2B can be found in S1 Data. **(C)** Cytological analysis of wild type and ∆*ykvU* at hour 5 of sporulation. Phase contrast, membrane staining, and cytoplasmic mCherry (red) in the mother cell and cytoplasmic CFP (green) in the forespore are shown. The phase-bright ring around the forespore and the gap between mother cell and forespore cytoplasm in the *ykvU* mutant are highlighted (yellow carets). Schematic of a sporulating cell with an enlarged intermembrane space is shown. Additional examples can be found in S2B Fig. Scale bar represents 1 μm.

Two well-characterized sporulation genes that our Tn-seq screen failed to identify were *racA* and *fisB* (Fig 2A). RacA acts early during sporulation to remodel the replicated chromosomes into an elongated structure called the axial filament and anchors the origins at opposite cell poles [32,33]. Despite the pronounced cytological phenotype in cells lacking RacA, the mutant is reduced only ~2-fold in sporulation efficiency. FisB functions in membrane fission, the final stage in the phagocytic-like process of engulfment [34]. In sporulating cells lacking FisB, the mother cell membranes migrate around the forespore but are delayed and impaired in scission and release of the forespore into the mother cell cytoplasm. Again, despite its dramatic cytological phenotype, the FisB mutant is reduced only ~4-fold in sporulation efficiency. The mild phenotypes of these mutants likely account for the recovery of transposon insertions in these genes at T24 (Fig 2A). We wondered whether we would have identified *racA* and *fisB* and additional genes in a more stringent sporulation screen that demanded timely differentiation into heat-resistant spores. Specifically, we hypothesized that transposon insertions in these genes delay development. To test this, we first assayed sporulation efficiency in wild-type cells in a time course after the onset of starvation and found that by hour 5, between 0.01% and 0.4% of the sporulating cells had differentiated into heat-resistant spores. We then repeated the Tn-seq screen but harvested and heat-selected the transposon library of sporulating cells at hour 5 (T5).

As anticipated, transposon insertions in *racA* and *fisB* were significantly under-represented at this early time point compared to time 0 and hour 24 (Figs 1C and 2A). Along with these two genes and 156 of the 158 genes we identified at T24, there were 147 additional genes in which transposon insertions delayed the formation of heat-resistant spores (Fig 1C and S2 Table and S1 Data). Out of the 15 previously identified sporulation genes that we failed to detect at T24, seven were hits at T5 (S2 Table). Interestingly, 36 of the genes identified at T5 were previously reported to be expressed under sporulation control but deletions were not found to have sporulation defects [20–25]. An additional 38 genes identified at T5 are predicted to have sporulation-specific promoters (S2 Table) [35]. To investigate whether any of these T5 hits were indeed required for timely differentiation, we chose eight for which transposon insertions were significantly underrepresented at T5 compared to T0 and T24 (Fig 2 and S2A Fig). This set included genes transcribed by σ^F^ (*yqhH*, *yyaC*), σ^E^ (*ykvU*, *ykvI*, *prpB*), and σ^G^ (*yrbG*, *ykoN*, *yqgE*). The roles of these genes in sporulation are unknown or poorly characterized [36,37] but based on transcriptional profiling, their expression appears to be largely under sporulation control. Wild-type and the null mutants were grown in sporulation medium and samples were removed at hour 5 and 24 after the onset of starvation and incubated at 80°C for 20 min to kill vegetative cells and sporulating cells that had not yet achieved heat-resistance. In these assays, the sporulation efficiency of wild type at hour 5 ranged from 0.01%–0.4%. By contrast, in seven out of the eight mutants examined, there was a significant (7–1,500-fold) reduction in heat-resistant spores at this early time point (Fig 2B and S1 Data). Importantly and consistent with our Tn-seq data, at hour 24 the spore titers of the mutants were similar to wild-type (Fig 2B and S1 Data). These data suggest that many of the sporulation-induced genes previously identified by expression profiling play a direct role in the efficient and/or timely differentiation into a dormant spore. Furthermore, these data raise the possibility that many additional genes predicted to have sporulation promoters are indeed induced during sporulation and similarly function in efficient spore formation.

To investigate whether these mutants were indeed delayed in spore maturation and at what stage, we analyzed one (∆*ykvU*) that had a strong sporulation defect at hour 5 by fluorescence microscopy. Cells lacking *ykvU* initiated sporulation at approximately the same time as wild-type but exhibited a marked delay in the formation of phase-grey and phase-bright spores (Fig 2C and S2B Fig). Strikingly, a subset of the sporulating cells in the ∆*ykvU* mutant had a novel phenotype that, to our knowledge, has never been reported previously. The space between the forespore and mother cell membranes was significantly larger than wild type (Fig 2C and S2B Fig). This "distended intermembrane space" was most apparent in merged images of cytoplasmic mCherry that reports mother cell gene expression under σ^E^ control and cytoplasmic CFP that shows forespore gene expression under σ^G^ control (Fig 2C and S2B Fig). A distinct gap separated these cytoplasmic signals. Furthermore, a phase-bright ring was detectable surrounding the forespore in these sporangia (Fig 2C and S2B Fig). The nature of this phase-bright material in the intermembrane space is not known and awaits further investigation (see below). All together, our data indicate that *ykvU* and likely many other genes identified in our screen play important roles in timely spore maturation.

### Tn-seq Identifies Transposon Insertions That Trigger Premature Sporulation

**Fig 3:**
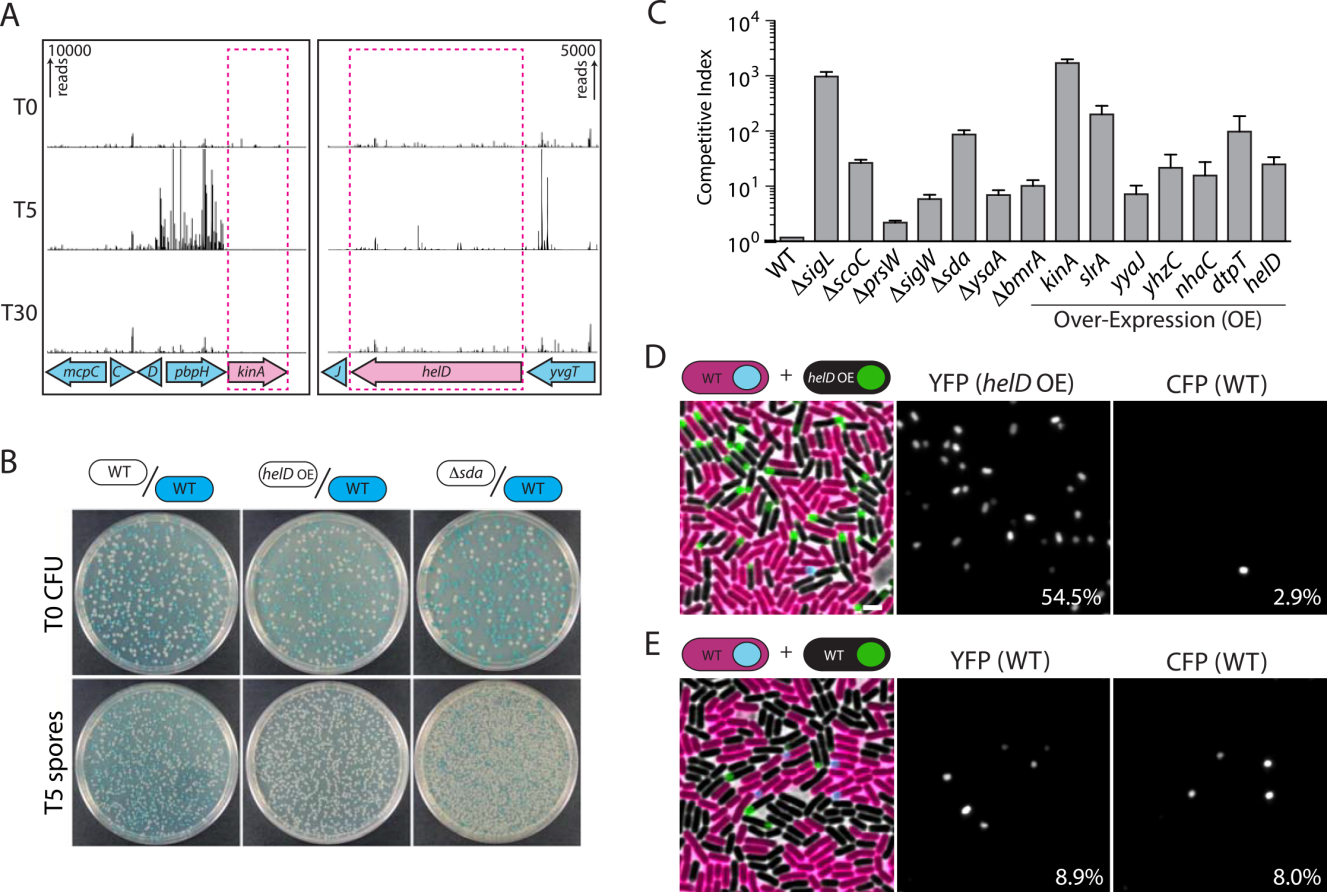
Tn-seq identifies mutants that initiate sporulation prematurely. **(A)** Transposon insertion profiles for two regions of the genome at T0, T5, and T24. The number of sequencing reads in these profiles is scaled 10–20 times higher than those in Figs 1 and 2. Insertions upstream of the *kinA* and *helD* genes are significantly enriched at T5. **(B)** Co-culture assay to assess premature spore formation of mutants identified by Tn-seq. Indicated strains were co-cultured at a 1:1 ratio with a wild-type (WT) strain constitutively expressing *lacZ*. At the onset of starvation (T0) and after heat-treatment at T5, cultures were plated on LB agar containing X-gal to determine the ratio of mutant to WT CFU. Representative examples of plates showing T0 CFU and T5 spores (heat-resistant CFU) from three mixed cultures (WT/WT, *helD* over-expression (OE)/WT, and Δ*sda*/WT) are shown. **(C)** Bar graph showing the competitive indices of indicated mutants and over-expression strains. The competitive index represents the ratio of mutant to WT spores at T5 divided by the ratio of mutant to WT CFU at T0. Error bars represent standard error of the mean from two biological replicates. The raw underlying numerical data for Fig 3C can be found in S1 Data. **(D)** A strain over-expressing *helD* (*helD* OE) initiates sporulation prematurely. The *helD* OE strain and WT constitutively expressing mCherry were mixed at a 1:1 ratio and cultured in sporulation medium. The cells were monitored by fluorescence microscopy for entry into sporulation as assayed by σ^F^ activity in the forespore. Both strains contain a σ^F^-responsive promoter (P_*spoIIQ*_) fused to *yfp* (*helD* OE) or *cfp* (WT). The percentage of *helD* OE and WT cells that have activated σ^F^ are indicated. More than 1,000 cells were counted for each time point. Scale bar indicates 3 µm. Additional examples can be seen in S3B Fig. **(E)** The experiment in (D) was performed using a 1:1 co-culture of two WT strains.

In addition to the delayed sporulation mutants described above, visual inspection of the insertion profiles from the Tn-seq screen at hour 5 revealed a second class of insertions. We identified 43 chromosomal regions (S3 Table) where transposon insertions were significantly overrepresented at this early time point in sporulation (Figs 1C, 3A and S3A). Overrepresentation suggests that insertions at these positions result in premature and/or accelerated sporulation. Mutants of this variety with a so-called “derepressed” sporulation phenotype were described in the 1960s and 1970s [38–40]. In most cases, the mutations responsible for these phenotypes were never defined genetically. The insertions in our Tn-seq screen fell into two classes: those that were upstream of a gene or operon and likely result in over-expression of the adjacent locus, and those that were inserted within a coding region and likely cause gene inactivation (S3 Table). Among the regions in the former class were transposon insertions upstream of *kinA* and *kinB* (and possibly *kinD*) three of the histidine kinases that activate the master sporulation transcription factor Spo0A (Fig 3A and S3 Fig) [41]. Previous work indicates that over-expression of *kinA* and *kinB* can trigger entry into sporulation in LB medium prior to nutrient exhaustion [42]. We suspect that the promoter for the spectinomycin resistance gene in the magellan6 transposon [43] similarly provides additional expression of these kinases. In support of this idea, transposon insertions were commonly found in non-essential genes in operons with essential genes downstream (S4 Fig). Insertions were also overrepresented upstream of the *opp* (formerly, *spo0K*) operon encoding an oligopeptide permease [44,45]. This transporter has been shown to import quorum-sensing oligopeptides that promote sporulation and competence [46–49]. Thus, cells over-expressing this permease likely initiate sporulation prematurely. Similarly, we identified insertions upstream of *dtpT* encoding a putative di- and tri-peptide permease [50], which we hypothesize can transport these oligopeptides or smaller unidentified signaling molecules. Moreover, insertions within the *scoC* gene were also overrepresented. ScoC is a transcriptional regulator that has been shown to repress expression of *opp* and *dtpT* [51,52]. Accordingly, in its absence, the level of these two permeases would increase. We also identified insertions in the *sda* gene, which encodes a cell-cycle regulator of KinA and KinB that prevents entry into sporulation during the initiation of replication [53,54]. Inactivation of *sda* could also result in premature entry into sporulation. The strong enrichment of transposon insertions upstream of and within genes that are known to function in the initiation of sporulation suggests that the enrichment of insertions adjacent to and within uncharacterized genes could define additional factors that control entry into this developmental process.

We selected 14 loci that were among the most overrepresented at T5 to validate our findings. Insertions at seven of these sites were predicted to cause gene inactivation (*sigL*, *scoC*, *prsW*, *sigW*, *sda*, *ysaA*, and *bmrA*); for these we used deletion mutants from the knockout collection. Insertions at the other seven loci were predicted to cause over-expression, and for these, we built IPTG-inducible promoter fusions to four (*slrA*, *yyaJ*, *nhaC*, and *yhzC*); we reconstructed transposon insertions upstream of two (*dtpT* and *helD*); and we used a previously characterized IPTG-inducible allele of *kinA* [42]. Each null mutant and over-expression strain (for simplicity, referred to as the mutants) was mixed at a 1:1 ratio with wild-type cells that constitutively expressed *lacZ*, and the two strains were co-cultured in sporulation medium. At the onset of starvation (T0), samples from the mixed cultures were removed and plated on LB agar containing X-gal to assess the ratio of mutant (white colonies) to wild-type (blue colonies) cells upon entry into sporulation (Fig 3B). At hour 5 (T5), samples were removed and incubated at 80°C for 20 min and the spores were plated on LB X-gal. The ratio of mutant to wild-type heat-resistant colony forming units (CFU) was compared to the ratio of CFU at T0 (Fig 3B). All of the strains that we tested had significantly (2–1,600-fold) more spores at T5 than wild type (Fig 3C and S1 Data). Importantly, virtually all the mutants (with the exception of Δ*sigL* and Δ*ysaA*) had growth curves that were indistinguishable from wild type (S5 Fig and S1 Data), suggesting that in most cases, early spore maturation was not a consequence of premature starvation. Thus, our Tn-seq screen identified a new class of sporulation genes whose inactivation or over-expression results in earlier spore maturation.

Finally, we chose four genes (*helD*, *nhaC*, *yhzC*, and *sigL*) to investigate whether their over-expression (or deletion in the case of *sigL*) resulted in premature entry into sporulation. These four genes were chosen based on the strength of their phenotypes and because they had not been previously implicated in sporulation. This analysis was performed in mixed cultures with wild-type cells that contained a constitutively expressed *mCherry* allele (P_*veg*_-*mCherry*) and a σ^F^-responsive promoter (P_*spoIIQ*_) fused to *cfp* to monitor an early stage in sporulation. The strains that overexpressed *helD*, *nhaC*, or *yhzC* or lacked *sigL* harbored the same σ^F^-responsive promoter fused to *yfp* (P_*spoIIQ*_-*yfp*). The mixed populations were grown in sporulation medium and then visualized at the onset of starvation and during the early stages of spore formation. By hour 1 after the onset of starvation, 4–10-fold more mutant cells had entered sporulation (yellow fluorescent protein [YFP] positive, mCherry negative) than wild type (cyan fluorescent protein [CFP] and mCherry positive) (Fig 3D and S3B Fig). Importantly, a mixed population of wild-type cells harboring the sporulation reporters with and without cytoplasmic mCherry had similar percentages of sporulating cells (Fig 3E). All together, these results suggest that most of the insertions sites identified by Tn-seq define genes that when over-expressed or inactivated promote premature initiation of sporulation.

### Tn-seq Screen Identifies 24 New Sporulation Genes

Our success in identifying most of the previously identified sporulation genes prompted us to investigate whether any of the uncharacterized genes identified in the Tn-seq screen represent new sporulation loci. To assess the sporulation phenotypes of the null mutants, we individually tested strains derived from the *B*. *subtilis* knockout collection for sporulation efficiency 24 h after the onset of exhaustion. For these experiments, we used null mutants for which the antibiotic cassettes had been excised to reduce the chance of polar effects. Among the 59 mutants tested, 24 were reduced in sporulation greater than 2-fold, 15 of these were reduced by more than 5-fold, and nine by 10-fold or more (Table 1). The majority of the 35 false-positive hits fell into two classes: (i) mutants that had a moderate sporulation defect (50%–80% compared to wild type) and may actually represent true sporulation genes and (ii) mutants in which insertions were underrepresented but far from eliminated at T24. All of these genes were also identified as hits at T5 (S2 Table). For comparison, we also performed sporulation assays on 30 of the 37 genes known to be required for sporulation that have not been extensively characterized. The sporulation efficiencies of these mutants are reported in Table 1. Collectively, our Tn-seq screen uncovered 24 new genes that are required for efficient sporulation. Among these, none have been reported to be under sporulation control suggesting that many also function during vegetative growth. However, 12 of these genes are predicted to have sporulation-specific promoters (S2 Table) [35], raising the possibility that they are induced during development.

### A Cytological Assay to Characterize the Sporulation Mutants

**Fig 4:**
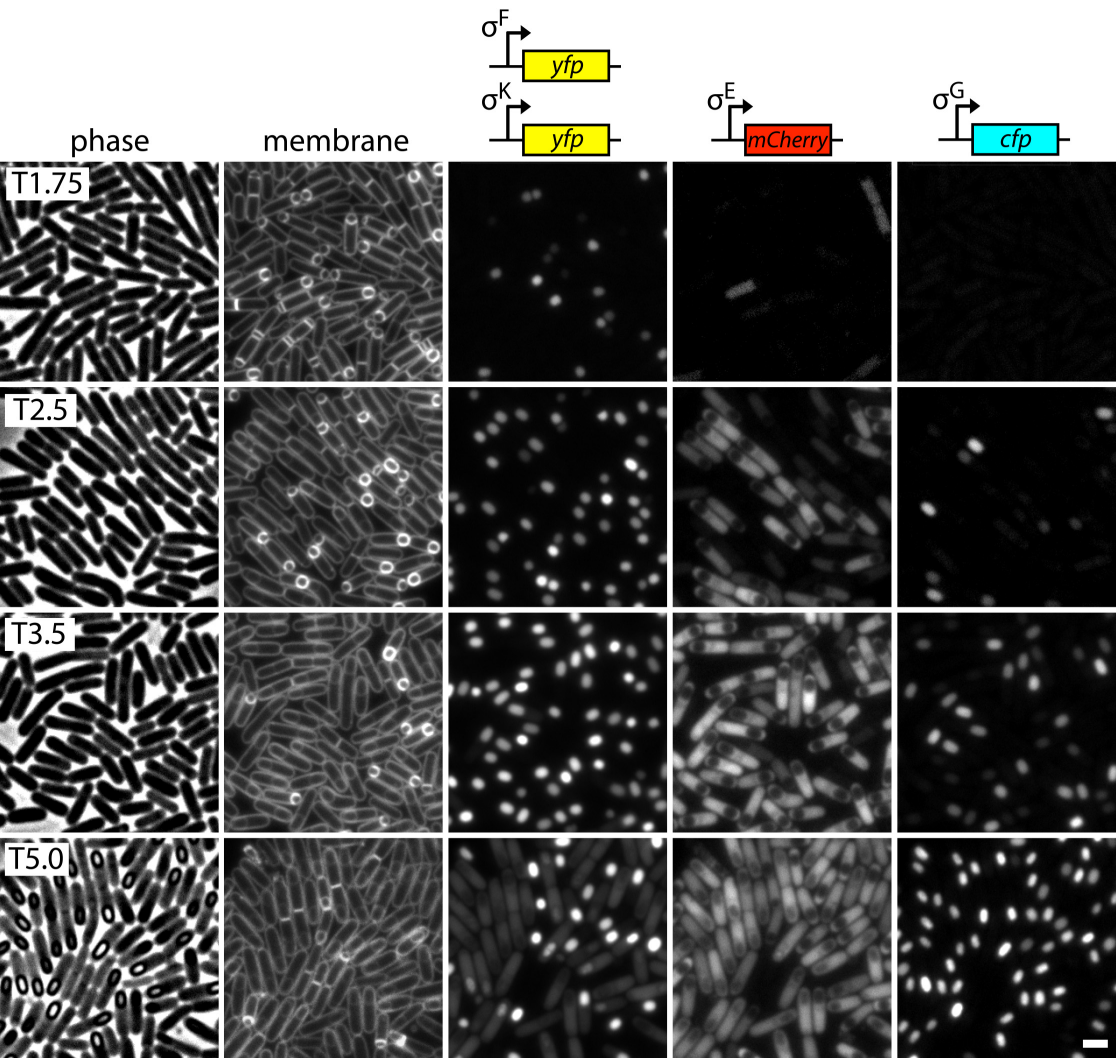
Cytological sporulation assay. Representative images of wild-type cells harboring four transcriptional fusions at hour 1.75 (T1.75), 2.5 (T2.5), 3.5 (T3.5), and 5 (T5) of sporulation. Phase contrast, membrane staining and the indicated fluorescent fusions are shown. Scale bar indicates 2 μm. At hour 5, sporangia with YFP in the mother cells produced under σ^K^ control have reduced σ^F^-directed YFP in the forespore but maintain forespore CFP under σ^G^ control.

To rapidly assess the stage at which these new sporulation mutants were impaired, we built a strain that reports on all four compartment- and stage-specific sigma factors (σ^F^, σ^E^, σ^G^, and σ^K^) (Fig 4). The reporter for the first compartment-specific transcription factor σ^F^ that is activated in the forespore was a promoter (P_*spoIIQ*_) fusion to *yfp* [55]. The P_*spoIID*_ promoter that is recognized by the first mother cell-specific transcription factor σ^E^ [56] was fused to *mCherry*. The reporter for the late-acting forespore transcription factor σ^G^ was a promoter (P_*sspB*_) fusion to *cfp* [57]. Finally, the P_*gerE*_ promoter, recognized by the late-acting mother cell transcription factor σ^K^ [58], was fused to *yfp*. σ^F^-dependent YFP fluorescence is first observed in the forespore at hour 1.5. By the time σ^K^ is activated in the mother cell (hour 4), σ^F^ activity is largely absent and YFP fluorescence is significantly reduced (Fig 4). Accordingly, we could use this fluorescent protein twice. For these experiments, we induced spore formation by re-suspending exponentially growing cells in minimal medium [59] to provide more synchronous sporulation. We monitored differentiation at four time points (hours 1.75, 2.5, 3.5, and 5) by phase contrast, membrane staining, and the four fluorescent reporters. As can be seen in Fig 4, in addition to the activity of the four stage- and compartment-specific transcription factors, this assay allowed us to assess polar division, engulfment, membrane fission, late spore development (phase grey), and spore maturation (phase bright).

### Cytological Characterization of the New Sporulation Mutants

**Fig 5:**
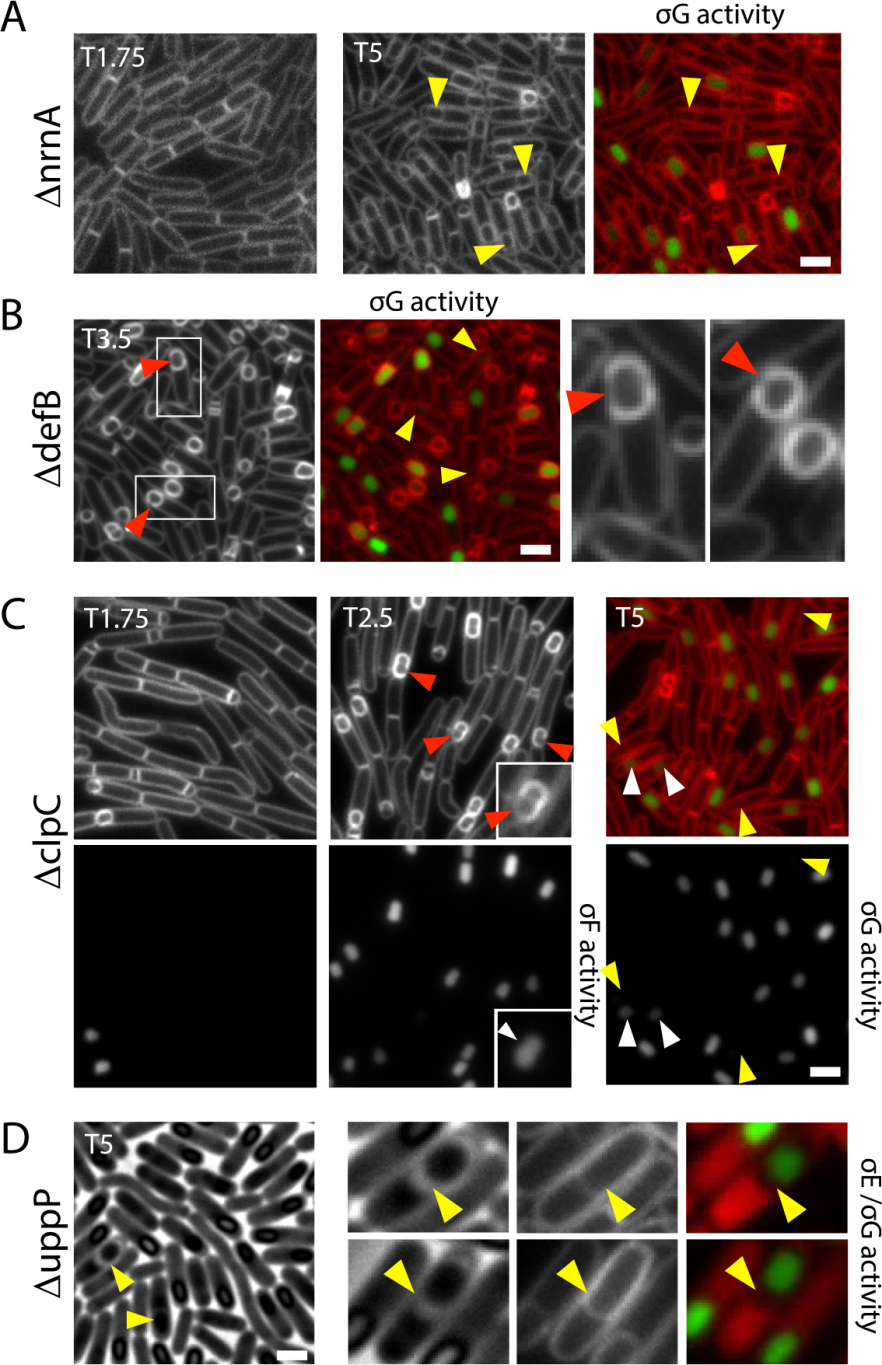
Cytological characterization of sporulation mutants reveals diverse phenotypes. **(A)** Representative images of the ∆*nrnA* mutant at hours 1.75 (T1.75) and 5 (T5) of sporulation. At T1.75, very few cells have polar septa indicative of impaired sporulation initiation. At T5, engulfed forespores with undetectable σ^G^ activity are highlighted (yellow carets). **(B)** Representative images of the ∆*defB* mutant at hour 3.5 (T3.5) of sporulation. Membrane fission is impaired compared to wild-type and a subset of engulfed forespores lack σ^G^ activity (yellow carets). Selected areas are enlarged to highlight asymmetric membrane migration around the forespore (red carets). **(C)** Representative images of the ∆*clpC* mutant at indicated time points. At T1.75, few cells have polar septa and a small number have σ^F^ activity. At T2.5, membrane defects during engulfment are highlighted with red carets and pinched forespores with white carets. At T5, small forespores with reduced (white carets) or undetectable (yellow carets) σ^G^ activity are indicated. **(D)** Representative images of the **∆***uppP* mutant at T5 of sporulation. Forespores with enlarged space between the outer and inner spore membranes are indicated (yellow carets). Scale bar is 2 μm. S6 and S7 Figs have representative images for all the other mutants tested.

Using our reporter strain we analyzed 21 of the newly identified sporulation genes as well as nine additional sporulation mutants that had been previously identified but not extensively characterized. The mutants were sporulated by resuspension in groups of six and monitored at the time points shown in Fig 4. Among the 30 mutants analyzed, ten had no discernable phenotype in our assay (Table 1 and S6 Fig). These mutants are either impaired at stages that were not assayed, have weak or subtle phenotypes, or are not required when cells are sporulated by resuspension. Of the remaining 20, 11 (*gidA*, *trmE*, *resA*, *yqhT*, *speE*, *gapB*, *smpB*, *rasP*, *nrnA*, *clpC*, and *minJ*) appeared to be impaired in sporulation intiation having many fewer cells with polar divisions and forespore-specific gene expression at early time points (Fig 5 and S7 Fig). Furthermore, six mutants (*gidA*, *ytxG*, *trmE*, *clpC*, *defB*, and *minJ*) had defects that were specifically revealed by membrane staining (Fig 5 and S7 Fig). These mutants displayed defects in polar division, membrane migration, and/or membrane fission. Several of these mutants also had subtle membrane abnormalities in predivisional cells, suggesting that similar pathologies exist during vegetative growth but are significantly enhanced during morphogenesis (S7 Fig). Six mutants (*yqzE*, *ydcC*, *yqfD*, *nrnA*, *clpC*, and *defB*) contained a subpopulation of sporulating cells in which σ^G^ activity was reduced or absent (Fig 5 and S7 Fig). Among these, many displayed a partially penetrant small forespore phenotype that correlates with defects in σ^G^ activity (Fig 5 and S7 Fig) (see below) [60,61]. Several of the mutants had distinct cytological phenotypes. All are described in Table 1 and the primary cytological data can be found in S7 Fig.

Below, we briefly highlight four genes (*nrnA*, *defB*, *clpC*, and *uppP*) that had representative or unusual mutant phenotypes (Fig 5). *nrnA* encodes a nanoRNase responsible for degradation of RNA fragments [62]. Cells lacking *nrnA* had an intermediate sporulation defect (5% of wild type) and were significantly delayed in the entry into sporulation. At hour 1.75, virtually no cells had polar septa (Figs 4 and 5A). All stages of differentiation analyzed were similarly delayed in this mutant. However, activation of σ^G^ appeared to be particularly impacted by the absence of *nrnA* (Fig 5A). During sporulation in wild-type cells, σ^G^ activation is coincident with the completion of engulfment (Fig 4). In the *nrnA* mutant, a substantial number of engulfed forespores had reduced σ^G^-dependent CFP fluorescence. Interestingly, some of these cells also appeared to have smaller forespores: a phenotype that correlates with reduced σ^G^ activity. The role of RNA degradation in entry into sporulation has not been actively explored but these findings raise the possibility of a novel regulatory pathway.

*defB* encodes a N-formylcysteine deformylase [63]. The mutant had a modest sporulation defect (9% of wild type) (Table 1). However, cytological analysis revealed an unusual engulfment phenotype in which the mother cell membranes appeared to migrate asymmetrically around the forespore meeting at non-polar positions (Fig 5B). In addition to and possibly a consequence of this uneven migration, membrane fission, the last step in engulfment, was delayed and impaired (Fig 5B). Furthermore, in a subset of cells, σ^G^ activity in the forespore was reduced or absent. The mechanism by which a deformylase impacts membrane migration is currently unknown; however, based on the strength of the cytological phenotype, further investigation is merited.

ClpC is the ATPase subunit of the ATP-dependent ClpC-ClpP protease [64]. Previous work indicates that ClpCP degrades the anti-σ^F^ factor SpoIIAB in the forespore helping to lock σ^F^ in its active state [65]. The ∆*clpC* mutant has a strong sporulation defect (0.4% of wild type); however, it had not been cytologically characterized. Interestingly, the null mutant displayed a collection of cytological phenotypes, including delayed entry, asymmetric engulfment, reduced or no σ^G^ activity and a concomitant small forespore phenotype (Fig 5C). These phenotypes suggest that the ClpCP protease plays an important role in post-translational control of a host of proteins during development.

*uppP* is predicted to encode a minor undecaprenyl pyrophosphate phosphatase involved in recycling the lipid carrier undecaprenyl phosphate [66,67]. Cells lacking *uppP* have a sporulation efficiency of 0.4%. The mutant entered sporulation normally but was delayed in the production of phase-grey and ultimately phase-bright spores (Fig 5D) suggesting a defect in assembly of the spore cortex and/or spore dehydration [68]. Strikingly, a subset of sporulating cells in this mutant had the same distended intermembrane space phenotype observed in the *ykvU* mutant (Figs 2C and 5D). Furthermore, a similar phase-bright ring was present around the forespore (Fig 5D). Although this novel phenotype has not been reported previously, we have now observed it in two separate mutants. Intriguingly, YkvU is a member of the multidrug/oligosaccharidyl-lipid/polysaccharide (MOP) exporter superfamily and a subset of the proteins in this family transport polysaccharide precursors linked to undecaprenyl phosphate across the membrane [69]. This provides a possible explanation for the shared phenotype. YkvU is produced in the mother cell under the control of σ^E^ and specifically localizes in the membranes surrounding the forespore [20,36,37]. In the absence of this putative flippase, an uncharacterized polysaccharide precursor would not be transported into the intermembrane space. Similarly, in the absence of UppP, recyling of the lipid carrier is predicted to be impaired, resulting in a reduction in the transport of this hypothetical precursor (S2C Fig). In the context of this model, one possible explanation for the distended intermembrane space in the mutants is that the polysaccharide produced in this putative pathway could help hold the two membranes surrounding forespore together. Dissecting the roles of these proteins and this unusual mutant phenotype awaits future investigation.

Finally, two new sporulation genes (*yqzE* and *ywmB*) were characterized in greater detail. These experiments defined two novel factors: one (SpoIIIL) helps maintain forespore differentiation and σ^G^ activity (see Box 1) and the other (SpoIIT) functions directly in the cell–cell signaling pathway that triggers proteolytic activation of σ^E^ in the mother cell (see below).

**Box 1. SpoIIIL Is Required for σ^G^ Activation**

**Fig 6:**
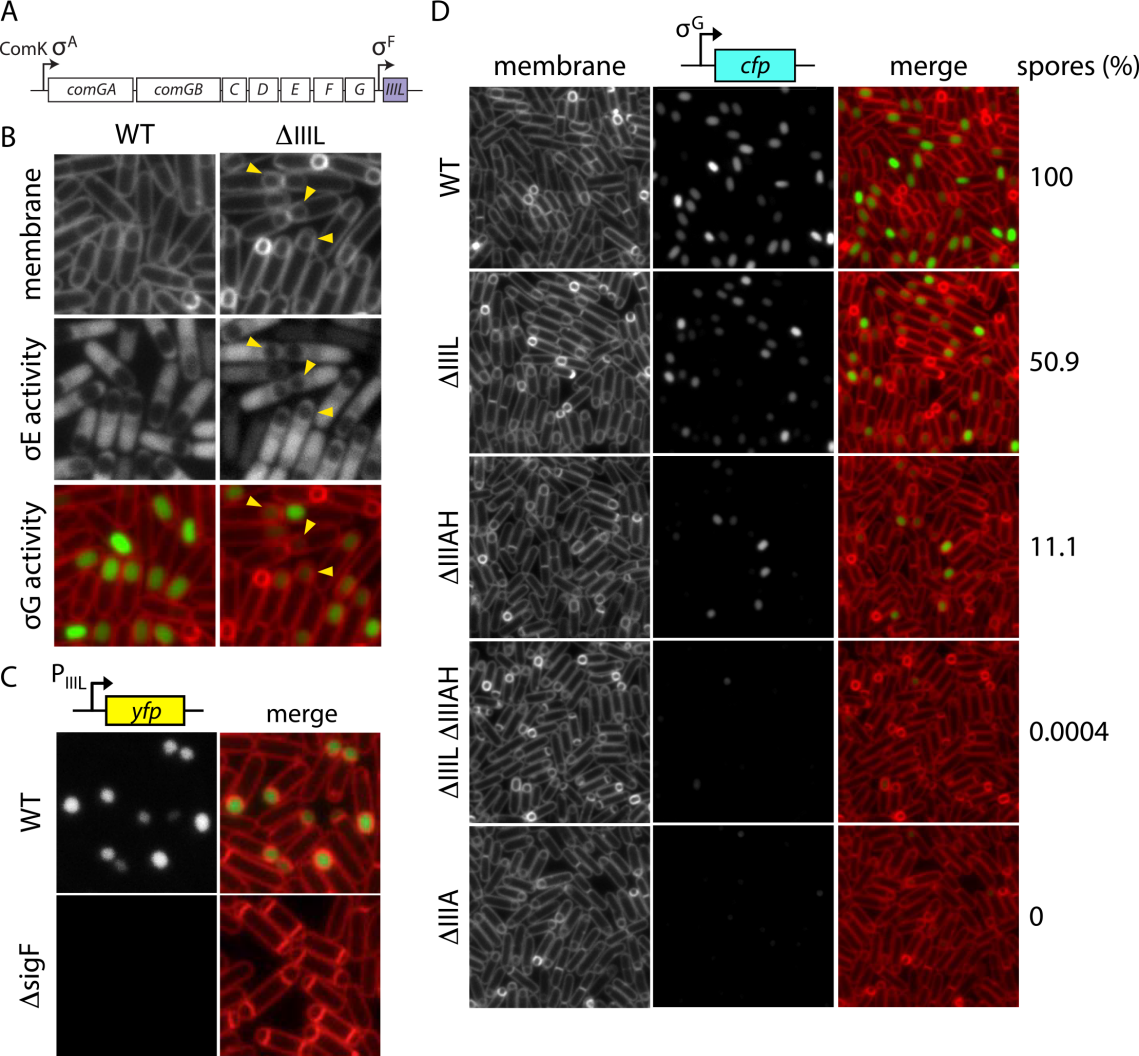
*SpoIIIL* is required for σ^G^ activation. **(A)** The *spoIIIL* genomic locus. **(B)** Representative images of the ∆*spoIIIL* mutant and WT at hour 3.5 of sporulation. Small forespores with reduced σ^G^ activity are indicated (yellow carets). The small forespores are also highlighted by the cytoplasmic mCherry signal in the mother cell reporting on σ^E^ activity. **(C)** Representative images of the intergenic region between *spoIIIL* and *comGG* (P_*spoIIIL*_) fused to *yfp* at hour 2 of sporulation. WT and a ∆*spoIIAC* (∆*sigF*) mutant are shown. **(D)** Synergistic defects in σ^G^ activity, forespore size, and sporulation efficiency in the ∆*spoIIIL* ∆*spoIIIAH* double mutant. Representative images at hour 3.5 of sporulation are WT, ∆*spoIIIL*, ∆*spoIIIAH*, the ∆*spoIIIL ∆spoIIIAH* double mutant and ∆*spoIIIA*. Spore titer relative to wild-type spores at hour 30 are indicated at the right. Scale bar is 2 μm.

Cells lacking *yqzE* (renamed *spoIIIL*) had a sporulation efficiency of 14% compared to wild type (Table 1). Cytological analysis of the mutant revealed that a subpopulation of sporulating cells had small forespores that often had reduced or no detectable σ^G^ activity (Fig 6B). These phenotypes are reminiscent of the small and collapsed forespores observed in cells lacking *spoIIQ* or any of the genes in the *spoIIIA* operon [60]. The forespore protein SpoIIQ and the eight SpoIIIA (spoIIIAA-AH) proteins produced in the mother cell assemble a transenvelope complex (referred to as the SpoIIIA-IIQ complex) that connects the mother cell and the developing spore [60,70,71]. This complex is required to maintain transcriptional potential in the forespore, including σ^G^ activity, and is also required for forespore development [60,72]. It is thought that the complex functions as a specialized secretion system, enabling the mother cell to transport proteins and/or metabolites into the forespore and thereby maintain forespore physiology [60,72,73]. To investigate whether SpoIIIL functions in the SpoIIIA-IIQ pathway, we took advantage of the ∆*spoIIIAH* mutant phenotype. Unlike all other members of the SpoIIIA-IIQ complex, cells lacking SpoIIIAH have a relatively mild sporulation defect (1%–10% sporulation efficiency compared to 0.01%–0.001% for mutants in any other component), and the engulfed forespores are a mixture of small and normal sizes (Fig 6B) [60]. If the function of SpoIIIL relates to that of the SpoIIIA-IIQ complex, then we would expect a ∆*spoIIIL ∆spoIIIAH* double mutant to display synergistic phenotypes, and this is exactly what we observed (Fig 6B). The double mutant had almost no detectable σ^G^ activity and virtually all forespores were of small size, comparable to a mutant lacking the entire *spoIIIA* operon. Furthermore, like the *spoIIIA* operon mutant, the ∆*spoIIIL ∆spoIIIAH* double mutant had a sporulation efficiency of 0.001%.

*spoIIIL* is the last gene in the *comG* operon (Fig 6A) that encodes components of the DNA uptake machinery involved in natural competence [74]. *spoIIIL* is a small gene predicted to encode a novel cytoplasmic protein of 43 amino acids. The *comG* operon, which is not required for sporulation, is expressed during competence development under the control of the transcriptional regulator ComK [75]. Since ComK is not known to be active during sporulation, we suspected there was an additional promoter controlling *spoIIIL*. Noirot and co-workers [35] predicted a sporulation-specific promoter just upstream of *spoIIIL* (Fig 6A) and we found that this region promoted expression of YFP in the forespore in a σ^F^-dependent manner (Fig 6C). Furthermore, the *spoIIIL* gene including this upstream region was sufficient to restore sporulation to the null mutant and largely restored sporulation and σ^G^ activity to the ∆*spoIIIAH* ∆*spoIIIL* double mutant (S8 Fig; S1 Data). Collectively, these results suggest that SpoIIIL is a component of the SpoIIIA-SpoIIQ complex on the forespore side. Its function in this transenvelope complex awaits future investigation.

### YwmB (SpoIIT) Functions in the Cell–Cell Signaling Pathway That Activates σ^E^

**Fig 7:**
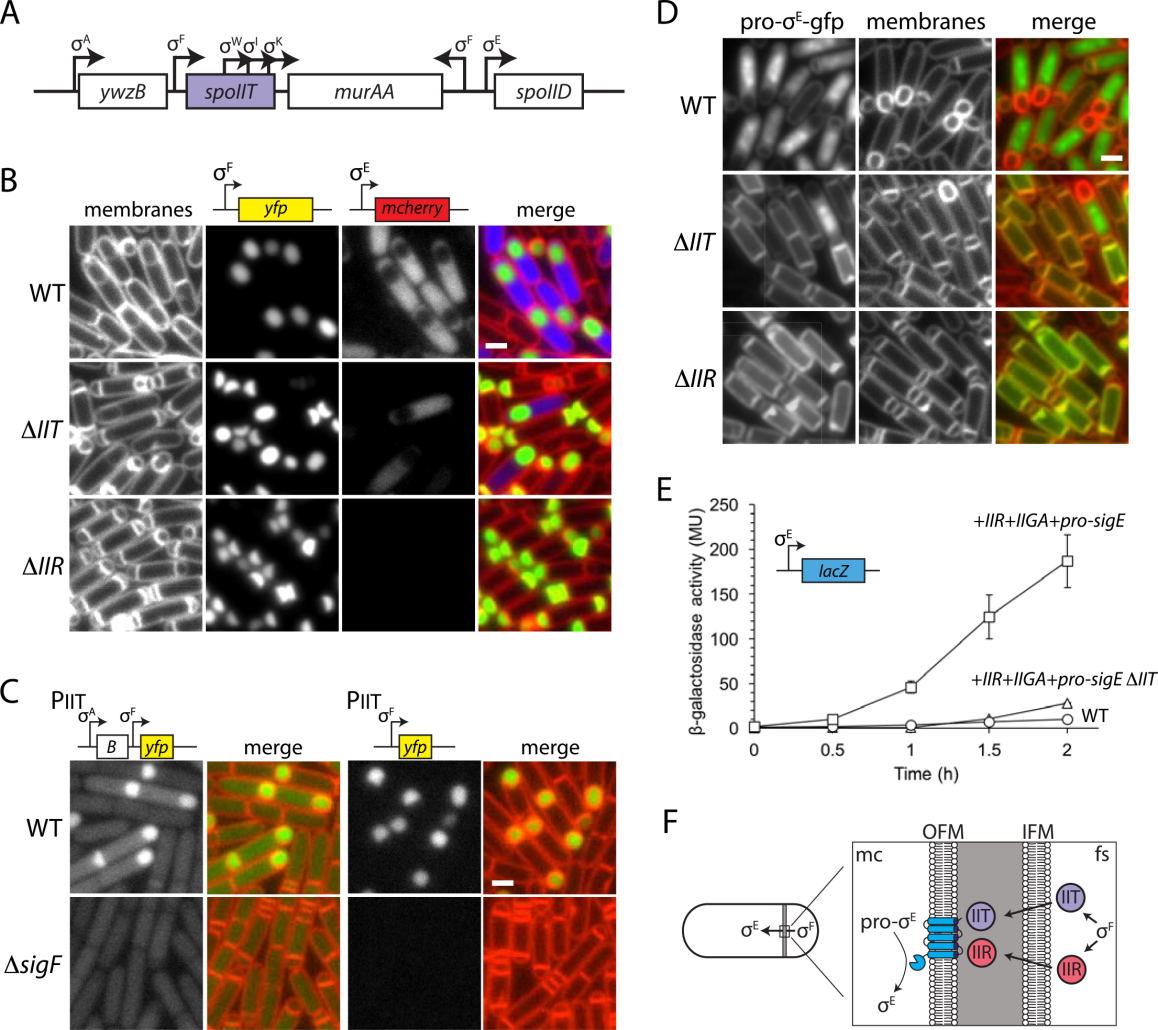
SpoIIT is required for efficient proteolytic processing of pro-σ^E^. **(A)** The *spoIIT* genomic locus. Three promoters for the essential gene *murAA* are predicted [35] to reside in the *spoIIT* coding sequence. **(B)** Cytological analysis of a nonpolar *spoIIT* deletion mutant that leaves the three promoters intact. Representative images of WT, ∆*spoIIT*, and ∆*spoIIR* mutants at hour 2 of sporulation. σ^F^ activity was monitored by P_*spoIIQ*_-yfp (green) and σ^E^ activity by P_*spoIID*_-mcherry (blue). Scale bar indicates 1 μm. Larger fields of sporulating cells and additional time points can be found in S9 Fig. **(C)** Transcriptional fusions identify a σ^F^-responsive promoter upstream of *spoIIT*. Two *spoIIT* promoter fusions to *yfp* are shown: one includes the upstream gene *ywzB* (labeled B) and its predicted σ^A^ promoter and the other contains 200 bp proximal to the *spoIIT* start codon. Expression from each fusion was examined in WT and a ∆*spoIIAC* (∆*sigF*) mutant at hour 1.5 of sporulation. **(D)** Localization of pro-σ^E^-gfp at hour 2 of sporulation in WT, ∆*spoIIT*, and ∆*spoIIR*. Pro-σ^E^-gfp is membrane-associated while σ^E^-gfp is cytoplasmic and nucleoid-associated. **(E)** SpoIIT is required for reconstitution of the pro-σ^E^ processing pathway in vegetatively growing cells. β-galactosidase assay monitoring σ^E^ activity (P_*spoIID*_-*lacZ*) in strains expressing *spoIIR spoIIGA*, and *pro-sigE* during vegetative growth in the presence and absence of *spoIIT*. As a negative control, a strain only expressing *spoIIGA* and *pro-sigE* was also tested. Expression of *spoIIGA/sigE/spoIIR* was induced by the addition of 500 μM IPTG to cells growing exponentially (OD 0.2) in rich medium (casein hydrolysate [CH]). At the indicated times after induction, a sample was removed and analyzed for β-galactosidase activity. Error bars represent standard error of the mean from two biological replicates. The raw underlying numerical data for Fig 7E can be found in S1 Data. **(F)** Model for the proteolytic activation of σ^E^ under σ^F^ control. SpoIIR (red) and SpoIIT (purple) are made in the forespore and secreted into the intermembrane space where they activate the processing enzyme SpoIIGA (blue).

Among the newly identified sporulation genes, the *ywmB* (renamed *spoIIT*) mutant had one of the most revealing phenotypes in our cytological assay. A large population of sporulating cells lacking SpoIIT had two polar divisions (referred to as an abortive disporic phenotype) and lacked σ^E^ activity (Figs 7B, S9 and S10A and S1 Data). Both phenotypes are hallmarks of a gene involved in the activation of σ^E^ [76]. This mother cell transcription factor is synthesized as an inactive membrane-associated precursor called pro-σ^E^ (Fig 7F) [77,78]. Proteolytic processing of pro-σ^E^ releases the mature and active transcription factor into the mother cell cytoplasm [79,80]. A signaling protein (SpoIIR) that is made in the forespore under the control of σ^F^ is secreted into the intermembrane space [81–83] and activates a protease (SpoIIGA) present in the septal membranes on the mother cell side (Fig 7F) [84–86]. Mutants in *spoIIR* or *spoIIGA* fail to activate σ^E^ resulting in abortive disporic sporangia (Fig 7B). The *spoIIT* mutant phenotype was not as penetrant as the ∆*spoIIR* and ∆*spoIIGA* mutants. At hour 2.5 of sporulation ~15% of the sporulating cells successfully activate σ^E^ and continue down the sporulation pathway (Figs 7B and S10A and S1 Data). Although SpoIIT is not absolutely required for σ^E^ activation, the identification of a new factor that might participate in this well-characterized signaling pathway prompted us to investigate SpoIIT further.

In our initial characterization of *spoIIT*, we used the insertion-deletion mutant from the *B*. *subtilis* knockout collection, which lacks the entire *spoIIT* coding sequence. This mutant had the strongest phenotype among all our newly identified sporulation genes, yielding no viable spores. However, the mutant could not be complemented by the *spoIIT* gene in trans. *spoIIT* is part of a three-gene operon (*ywzB*-*spoIIT*-*murAA*) (Fig 7A) and we wondered whether the antibiotic resistance gene that replaced *spoIIT* was causing inappropriate expression of *murAA*, leading to the sporulation defect. We attempted to remove the erythromcyin (*erm*) resistance cassette using Cre/*loxP*-mediated recombination [87], but were unable to excise it. There are three predicted promoters [35] within the *spoIIT* gene (Fig 7A) that could contribute to expression of the essential *murAA* gene, and in the insertion-deletion mutant, the *erm* promoter might provide this expression. Accordingly, we generated an in-frame deletion of *spoIIT* that removes the first 63 amino acids of the *spoIIT* coding sequence (including its predicted signal peptide) but retains all three promoters within the *spoIIT* gene. This mutant was viable and exhibited a more mild but reproducible sporulation defect (38% of wild type). Importantly, similar to the original mutant, it produced a high number of abortive disporic sporangia and failed to efficiently activate σ^E^ (Figs 7B, S9 and S10A and S1 Data). Furthermore, the mutant could be complemented by expression of *spoIIT* from an ectopic locus (S10B Fig). Thus, *spoIIT* is a *bona fide* sporulation gene and is required for efficient activation of σ^E^. The nonpolar *spoIIT* deletion was used for all the characterization presented in Fig 7, S9 Fig and S10 Fig and described below.

First, we investigated whether SpoIIT functions in the proteolytic activation of σ^E^. To do this, we examined a pro-σ^E^-gfp fusion during sporulation by fluorescence microscopy [80]. The pro-domain of σ^E^ is amphipathic resulting in membrane localization. Proteolytic processing releases mature σ^E^-gfp into the cytoplasm and upon association with core RNA polymerase, it co-localizes with the nucleoid. Wild-type, ∆*spoIIT*, and ∆*spoIIR* mutants harboring a functional pro-σ^E^-gfp fusion were induced to sporulate by resuspension and were examined by fluorescence microscopy (Fig 7D). Consistent with the idea that SpoIIT is required for efficient pro-σ^E^ processing, in most sporulating cells at hour 2, pro-σ^E^-GFP remained membrane-associated in the ∆*spoIIT* mutant (Fig 7D). Immunoblot analysis of pro-σ^E^ processing from sporulating cells (S10C Fig), provided further support for this conclusion.

The *spoIIT* gene is predicted to encode a secreted protein expressed under the control of the housekeeping sigma factor σ^A^ (Fig 7A). If SpoIIT functions, as our data suggest, in the activation of the pro-σ^E^ processing enzyme SpoIIGA it must act in the intermembrane space (Fig 7F). Because secretion into the intermembrane space from the mother cell side is thought to be inefficient [88], we wondered whether SpoIIT, like the signaling protein SpoIIR, is produced in the forespore. Although *spoIIT* is not predicted to have a sporulation-specific promoter [35], we constructed *yfp* fusions to the genomic regions upstream of *spoIIT* (Fig 7C) and analyzed compartment-specific expression during sporulation. A 774 bp fragment that encompased the σ^A^ promoter, the *ywzB* gene, and the intergenic region between *ywzB* and *spoIIT* fused to *yfp* displayed modest YFP fluorescence in non-sporulating and predivisional cells (Fig 7C). Strikingly, in sporulating cells, strong forespore-specific YFP fluorescence was observed (Fig 7C). A 360 bp fragment that contained part of *ywzB* gene and the intergenic region between *ywzB* and *spoIIT* exclusively displayed forespore-specific gene expression. Furthermore, forespore-specific YFP fluorescence from both reporters required σ^F^ (Fig 7C). These data indicate that *spoIIT* is expressed at low levels during vegetative growth but is induced to higher levels in the forespore under the control of σ^F^. Thus, SpoIIT and SpoIIR are both produced in the forespore and likely secreted into the intermembrane space. Interestingly, the *spoIIT* gene is located at -37° on the *B*. *subtilis* chromosome, in a region that, like *spoIIR*, is trapped in the forespore at the time of polar division [89]. Thus, both genes are transcribed as soon as σ^F^ is activated, ensuring timely activation of σ^E^ in the mother cell [90,91].

Finally, to address whether SpoIIT acts directly in the σ^E^ signaling pathway, we took advantage of an observation made over two decades ago in which cells artificially engineered to express pro-σ^E^, SpoIIGA, and SpoIIR during vegetative growth were found to trigger pro-σ^E^ processing and induce σ^E^ activity (as assayed by a σ^E^-responsive promoter (P_*spoIID*_) fused to *lacZ*) [81,83]. In other words, SpoIIR-SpoIIGA signaling could be reconstituted in the absence of sporulation. Since *spoIIT* is itself expressed vegetatively (Fig 7C), to assess its impact on σ^E^ activation in this reconstituted system, we compared σ^E^-dependent β-galactosidase activity in the presence and absence of SpoIIT (Fig 7E and S1 Data). In support of the idea that SpoIIT is directly involved in this signaling pathway, σ^E^ activity was markedly reduced in its absence. Collectively, these data support a model in which SpoIIT functions as a co-activator with SpoIIR in triggering SpoIIGA-dependent processing of pro-σ^E^. Bioinformatic analysis indicates that SpoIIT, like SpoIIR, is present in virtually all endospore formers (S11 Fig), lending further support to the idea that both proteins are part of a core signaling pathway.

## Discussion

Transposon-sequencing and other variations of signature-tagged mutagenesis [43,92–94] were originally developed to study commensal and pathogenic bacteria. In most cases, the molecular genetic tools in these organisms are less developed than classical models like *Escherichia coli*, *B*. *subtilis* and *Caulobacter crescentus*, and the benefits of these approaches were immediately apparent. This study, among others [95–98], highlights the utility of Tn-seq for defining gene function in well-studied model bacteria. By combining Tn-seq and the recently completed ordered null mutant collection, we have identified and validated over 20 new genes that are required for spore formation, uncovered phenotypes for many uncharacterized sporulation-induced genes, and discovered a novel set of loci that initiate development prematurely. In a complementary approach, Koo and Gross directly screened the ordered knockout library for sporulation mutants and identified an overlapping set of genes (personal communication). Tn-seq could similarly be used to identify novel genes required for other aspects of bacterial physiology, including natural competence, stationary phase survival, swimming, swarming, cell envelope stress, and DNA damage. Driven by Tn-seq and complemented by whole-genome sequencing, we are entering a renaissance in classical genetics of model organisms. When combined with ordered deletion collections, the genetic relationships suggested by these approaches can be systematically validated and explored with unprecedented speed.

### Outlook

The Tn-seq screens performed in this study expanded the number of genes required for sporulation to >170, but we anticipate that there are many additional factors yet to be discovered. Some of these missing genes are likely to be “condition-specific,” while others were probably missed due to limitations of the approach. Our screens were performed in Difco Sporulation Medium (DSM) in aerated shaking flasks. We suspect that there are genes specifically required for sporulation in media of different composition, on solid surfaces, or at the air–water interface in the context of a biofilm community. Other genes might be specifically required for sporulation within a polymicrobial community. Tn-seq opens the door to identify these condition-specific sporulation factors. Furthermore, a Tn-seq survey of this variety has the potential to define a core set of genes required for spore formation under all conditions. An important limitation of the Tn-seq approach is its inability to identify sporulation genes that are essential for viability, as insertions in these genes would be absent from our reference (T0) dataset. Similarly, small sporulation genes for which there were a limited number of transposon insertions could have been missed in our screens. Finally, an additional limitation is the inability to identify sporulation genes with redundant functions. Transposon insertions in any one of the redundant genes would not have a sporulation defect and would therefore not have been underrepresented among the heat-resistant spores. By building transposon libraries in mutants with mild or undetectable sporulation phenotypes, Tn-seq could be used to identify synthetic sporulation defects. Such an approach has the potential to unmask a large collection of functionally redundant sporulation factors [27].

Our study also provides important lessons related to the recently completed ordered knockout collection. The antibiotic cassettes used to generate the mutants in this library lack transcriptional terminators to ensure expression of genes in operons. However, by avoiding one polar effect, a new one may have been introduced: over-expression of downstream genes. The contribution of over-expression to particular phenotypes can be easily assessed by Cre-mediated excision of the antibiotic cassette. Our analysis of *spoIIT* revealed an additional way in which null mutants can generate phenotypes. The *spoIIT* coding region contains three predicted promoters that are all absent in the knockout. Their absence could explain the strong sporulation phenotype of the original mutant and/or our inability to excise the antibiotic cassette. Although complementation remains the gold standard for defining the genetic basis of a phenotype, promoter predictions [35] will help inform decisions about whether or not more precise deletions should be constructed, as was done in our study.

### New Sporulation Genes

Among the new mutants we identified that impaired spore-formation, the largest class were those that impacted the timing and/or efficiency of initiation. Many inputs must be integrated in the decision to enter this developmental pathway, including nutritional status, cell-cycle stage, environmental cues, and quorum signals. Inactivation of individual genes in one of these pathways is likely to have a relatively modest impact on sporulation efficiency. This could account for why many of these mutants have been missed in classical genetic screens. Furthermore, since many of these genes are unlikely to be under sporulation control, they would not have been implicated in sporulation based on transcriptional profiling. Some of the mutants we identified likely impair some aspect of growth or metabolism that only indirectly impacts initiation of sporulation. However, we suspect others will provide insights into the cues that are sensed and integrated during starvation.

Interestingly, the other large class of mutants we discovered impacted the activity of the late-acting forespore transcription factor σ^G^. σ^G^ becomes active after the completion of engulfment, when the forespore resides as a membrane-bound protoplast in the mother cell cytoplasm and is no longer in direct contact with the extracellular environment. Recent work suggests that the loss of σ^G^ activity in *spoIIIA* and *spoIIQ* mutants reflects a loss or reduction in metabolic activity of the spore [60,72]. These mutants have reduced transcriptional potential, are smaller in size, and appear to collapse. In light of these findings, we speculate that the large collection of mutants containing a subpopulation of cells with small forespores and impaired σ^G^ activity reflects the physiologically sensitive state of the forespore at this stage in development. After engulfment is complete, the forespore likely relies on nutrients from the mother cell and catabolism of its own macromolecules to support its developmental program and its differentiation into a dormant spore. Accordingly, mutants impaired in σ^G^ activity could be affecting spore metabolism and therefore represent a particularly interesting and unexplored class of genes for future analysis.

### Developmentally Delayed Mutants

One of the most exciting discoveries to emerge from our screens is the identification of 36 uncharacterized sporulation-induced genes whose inactivation appears to delay spore maturation. Many of these genes were deleted and tested for sporulation efficiency when they were originally identified [20,21,23], and almost all lacked phenotypes. Others were tested in competition experiments subjecting mixed populations to rounds of sporulation and germination [26]. Our data suggest that many of these genes have quantifiable phenotypes that can be easily assayed by examining sporulation efficiency at early time points. The specific defect in spore maturation can then be assessed by fluorescence microscopy either alone or in mixed culture (Figs 2C and 3D). The identification of known sporulation genes like *racA* and *fisB* as delayed mutants, coupled with the discovery of a novel phenotype for the *ykvU* mutant, suggests that a systematic analysis of the remaining mutants could uncover additional cytological phenotypes reflecting novel steps in spore morphogenesis. In addition, 42 genes that are predicted to have sporulation-specific promoters that have not been experimentally validated were also identified as delayed mutants. Our analysis raises the possibility that many of these genes could be specifically induced during spore formation. We suggest that transposon-sequencing can serve as a powerful complement to expression profiling and computational predictions in defining the complete sets of regulons.

### Premature Initiators

Finally, the discovery of transposon insertions that promote premature sporulation was an unexpected outcome of the Tn-seq screen and exemplifies the power of this approach. As discussed above, entry into sporulation requires the integration of multiple inputs, and mutants deficient in any one of them frequently exhibit only modest defects in sporulation. However, inappropriate overstimulation of one of these pathways has the potential to induce premature sporulation, leading to the observed “accelerator” phenotype in our Tn-seq screen. Thus, characterization of these genes could provide insight into the triggers for entry into spore maturation. Interestingly, several of the genes predicted to cause premature sporulation due to over-expression encode transporters raising the possibility that uptake of uncharacterized molecules in the medium could induce early sporulation. Furthermore, we showed that premature initiation could be elicited by over-expression of the *helD* gene, encoding a DNA helicase that increases transcriptional cycling [99]. Evidence suggests that HelD is involved in transcriptional adaptation to new environmental conditions and we propose that increased expression of HelD promotes more rapid induction of the transcriptional programs associated with sporulation. Finally, mutants in envelope stress pathways, in arginine utilization and phenylalanine metabolism all appear to trigger premature sporulation suggesting certain pathways and metabolites could serve as control points for monitoring starvation or nutrient limitation.

### Two Forespore Signals

Finally, we turn to the pro-σ^E^ processing pathway and our discovery that a second secreted protein produced in the forespore promotes the proteolytic activation of σ^E^ in the mother cell. Collectively, our analysis supports a model in which both SpoIIR and SpoIIT function as signaling molecules. Although it remains unclear how these proteins trigger SpoIIGA activity, both genes are present in virtually all endospore formers, suggesting important complementary roles. Interestingly, the second and later-acting signal transduction pathway between the forespore and mother cell similarly utilizes two signaling proteins. In this case, σ^G^ directs the synthesis of two secreted proteases in the forespore: SpoIVB and CtpB [100,101]. These proteases in turn activate an unrelated membrane metalloprotease that processes pro-σ^K^ in the mother cell [102,103]. SpoIVB is the primary protease, which cleaves a negative regulator of the pro-σ^K^ processing enzyme [104]. CtpB further proteolyzes this negative regulator ensuring efficient and robust activation of σ^K^ [105]. SpoIVB, like SpoIIR is essential for σ^K^ activation, while cells lacking CtpB are delayed but not blocked in σ^K^ activation [106]. Although neither SpoIIR nor SpoIIT are proteases, by analogy to the pro-σ^K^ processing pathway, we hypothesize that SpoIIR triggers SpoIIGA proteolytic activity and SpoIIT helps maintain SpoIIGA in its active state. In vitro reconstitution of this signaling pathway will provide a clearer picture of the role of both of these signaling molecules in activating pro-σ^E^ processing.

## Methods

### General Methods

*B*. *subtilis* strains were derived from 168 [107] or PY79 [108]. Unless otherwise indicated, cells were grown in LB or CH medium at 37°C. Sporulation was induced by resuspension at 37°C according to the method of Sterlini-Mandelstam [59] or by nutrient exhaustion in supplemented DS medium (DSMcomplete) [109]. Sporulation efficiency was determined in 24–36 h cultures as the total number of heat-resistant (80°C for 20 min) colony forming units (CFUs) compared with wild-type heat-resistant CFUs. β-galactosidase assays were performed as described previously [102]. Insertion-deletion mutants come from the BKE knockout collection or were generated by isothermal assembly [110] of PCR products followed by direct transformation into *B*. *subtilis*. All BKE mutants were back-crossed into *B*. *subtilis* 168 before assaying and prior to antibiotic cassette removal. Antibiotic cassette removal was performed using a temperature-sensitive plasmid that constitutively expresses Cre recombinase [87]. Tables of strains (S4 Table), plasmids (S5 Table) and oligonucleotide primers (S6 Table) and a description of strain and plasmid constructions can be found in S1 Methods.

### Transposon Insertion Sequencing

Transposon insertion sequencing (Tn-seq) was performed on two independently generated libraries as described previously [43,87,97]. Briefly, Magellan6x transposon library DNA was transformed into competent *B*. *subtilis* 168 cells. ~750,000 transformants were pooled, aliquoted, and frozen. An aliquot was thawed, washed in DSM, and diluted into 50 ml DSM at an OD600 of 0.05. Samples were harvested at the onset of starvation (T0) and 5 (T5) and 24 (T24) h later. The T5 and T24 samples were incubated at 80°C for 20 min, and plated on LB agar. ~750,000 colonies from germinated spores from each were pooled. Genomic DNA was extracted from all three samples and digested with MmeI, followed by adapter ligation. Transposon-chromosome junctions were amplified in 16 PCR cycles. PCR products were gel-purified and sequenced on the Illumina HiSeq platform using TruSeq reagents (Tufts University TUCF Genomics facility). Raw sequence reads are available in the Sequence Read Archive (Accession: SRP066259). Reads were mapped to the *B*. *subtilis* 168 genome (NCBI NC_000964.3), tallied at each TA site, and genes in which reads were statistically underrepresented were identified using the Mann Whitney *U* test. Visual inspection of transposon insertion profiles was performed with the Sanger Artemis Genome Browser and Annotation tool. To identify genomic regions (including inter-genic regions) in which reads were statistically overrepresented, we performed a sliding window Mann Whitney *U* test using a 13 TA site window size across all TA sites which register at least one read in both the T0 and T5 experiments. Regions that had a *p*-value ≤ 0.02 and were at least 3-fold enriched in T5 reads compared to T0 reads are listed in S3 Table.

### Fluorescence Microscopy

Sporulating cells were concentrated by centrifugation at 8 krpm for 1 min and immobilized on 2% agarose pads. Fluorescence microscopy was performed using an Olympus BX61 microscope equipped with a UplanF1 100X phase contrast objective and a CoolSnapHQ digital camera (Photometrics) or a Nikon TE2000 inverted microscope with a Nikon CFI Plan Apo VC 100X objective. Images were acquired using Metamorph software. Membranes were stained with TMA-DPH (50 μM) (Molecular Probes) and fission of mother cell membranes was assessed as previously described [34]. Exposure times were 400 ms for TMA-DPH and mCherry, 200 ms for YFP and CFP, and 1,000 ms for σ^E^-gfp. Image analysis and processing were performed in Metamorph.

### Immunoblot Analysis

Whole-cell lysates from sporulating cells were prepared as previously described [111]. Samples were heated for 5 min at 65°C prior to loading. Equivalent loading was based on OD_600_ at the time of harvest. Samples were separated on a 12.5% polyacrylamide gel and transferred to a PVDF membrane. Membranes were blocked in 5% nonfat milk with 0.5% Tween-20 for 1 h. Blocked membranes were probed with anti-σ^E^ (1:100) [112], anti-σ^F^ (1:5000) [65], anti-His (1:4000) (Genscript), or anti-σ^A^ [113] primary antibodies diluted into PBS with 0.05% Tween-20 at 4°C overnight. Primary antibodies were detected with horseradish-peroxidase conjugated anti-mouse or anti-rabbit antibodies (BioRad) and detected with Western Lightning ECL reagent as described by the manufacturer.

## Supporting Information

S1 Data Raw numerical values for Tn-seq analysis, sporulation efficiency measurements, gene expression, and image quantitation. (XLSX)

S1 Fig Tn-seq identifies known sporulation genes. Examples of transposon insertion profiles for ten known and “named” sporulation loci. Transposon insertions at the onset of starvation (T0) and after 24 h (T24) of sporulation are displayed for nine regions of the genome. The height of each vertical line reflects the number of sequencing reads at this position. “VT” refers to *spoVT*, “CB” to *spoIVCB*, and “IIIC” to *spoIIIC*.
(PDF)

S2 Fig Developmentally delayed sporulation mutants identified by Tn-seq. **(A)** Examples of transposon insertion profiles for six mutants delayed in sporulation. Transposon insertions at the onset of starvation (T0) and after 5 (T5) or 24 (T24) h of sporulation are displayed for six regions of the genome. **(B)** Additional examples of the Δ*ykvU* mutant phenotype as described in Fig 2B. Phase contrast, membrane staining and overlays of the mother cell (red) and forespore (green) cytoplasmic fluorescent proteins. Yellow carets highlight phase bright rings, the enlarged outer forespore membrane, and the gap between the two compartments. **(C)** Model for the functions of YkvU and UppP during sporulation. YkvU is a putative flippase produced in the mother cell that could transport lipid-linked precursors into the intermembrane space to synthesize an unidentified polysaccharide. UppP is an undecaprenyl pyrophosphate (UPP) phosphatase potentially involved in the recycling of the undecaprenyl pyrophosphate (UP) lipid carrier.
(PDF)

S3 Fig Transposon insertions enriched at hour 5 of sporulation. **(A)** Examples of transposon insertion profiles for 13 regions of the genome at the onset of starvation (T0) and after 5 (T5) or 24 (T24) h of sporulation. The scale of maximum reads is 10,000 (or 3,000 where indicated) to highlight the enrichment of reads at T5. The genes colored pink below the insertion profiles are predicted to be the one impacted by the insertions. For *yhaJ*, *yhaI*, *scoC*, *ysaA*, *prsW*, *sigW*, *sda*, *yqeF*, *bmrA*, and *sigL*, the insertions are predicted to cause gene inactivation. For *opp*, *kinB*, *slrA*, *sigH*, *dtpT*, and *yyaJ*, the insertions are predicted to cause over-expression. **(B)** Premature initiation of sporulation in the Δ*sigL* mutant and strains over-expressing (OE) *yhzC* and *nhaC*. All three strains harbor a P_*spoIIQ*_-*yfp* fusion to monitor and early stage of sporulation. Strains were separately mixed 1:1 with wild type (WT) harboring constitutively expressed mCherry and P_*spoIIQ*_-*cfp*. Images are from hour 1 after the onset of starvation. The percentage of sporulating cells (as assayed by σ^F^ activity) in the mutant (YFP positive, mCherry negative) and the WT (CFP positive, mCherry positive) are shown for each culture.
(PDF)

S4 Fig Magellan6 insertions are non-polar. Transposon insertion profiles for four regions of the genome in which an essential gene (pink) resides downstream of a nonessential gene (blue) in an operon. The recovery of insertions in the nonessential blue gene provides evidence that the transposon can promote expression of the downstream essential gene. The profiles depicted are derived from cells harvested at the onset of starvation.
(PDF)

S5 Fig Most mutants that sporulate prematurely enter stationary phase in a manner similar to wild type. Growth curves of all validated prematurely sporulating mutants. Indicated mutants and over-expressing (OE) strains are shown in comparison to WT. For each growth curve, the WT is depicted in black and the mutant in red. Δ*sigL* and Δ*ysaA* mutants are the only strains that enter stationary phase earlier than WT. The raw underlying numerical data for S5 Fig can be found in S1 Data.
(PDF)

S6 Fig Sporulation mutants with no cytological phenotypes through hour 5 of sporulation. Representative images of ten mutants harboring four transcriptional fusions at hour 5 (T5) of sporulation. Phase contrast, membrane staining and the indicated fluorescent fusions are shown. Scale bars are 2 μm.
(PDF)

S7 Fig Additional sporulation mutants with cytological phenotypes. Representative images of 14 mutants harboring four transcriptional fusions at the indicated times after the initiation of sporulation **(A)** At hour 1.75 (T1.75), the ∆*gapB* mutant has few polar septa indicative of a delay in entry into sporulation. At hour 5 (T5), very few cells have completed engulfment and most forespores lack detectable σ^G^ activity. **(B)** The **∆***resA* mutant at T1.75 has few cells with polar septa and a small number with σ^F^ activity indicative of an early delay in sporulation. At hour 5 (T5), many cells have completed engulfment but most have not become phase-grey. **(C)** The **∆***yqhT* mutant at T1.75 has many cells with polar septa but relatively few with σ^F^ activity. At T5, many cells have completed engulfment but most have not become phase-grey. **(D)** The ∆*yqfD* mutant at hour 5. A subset of sporulating cells have small forespores (white carets) and reduced σ^G^ activity (yellow carets). The two phenotypes are not always correlated in this mutant. Enlarged examples of small forespores and forespores with reduced σ^G^ activity are shown on the right. **(E)** The ∆*speE* mutant at T1.75 has few cells with polar septa and almost no cells with σ^F^. At T5, many cells have completed engulfment but most have not become phase-grey. **(F)** The ∆*smpB* mutant at T1.75 has few cells with polar septa and no σ^F^ activity. At T5, most cells have completed engulfment but no forespores are phase-grey. **(G)** The ∆*minJ* mutant at T1.75 has few cells with polar septa. At T2.5, some of the engulfed forespores lack σ^F^ activity. There are a few large cells with strongly staining membranes that have σ^F^ activity (inset). By T5, some spores are phase bright but many sporulating cells have arrested or lysed. **(H)** The ∆*pgcA* mutant and the ∆*gtaB* mutant at hour 5 have relatively normal-sized forespores and small mother cells. These phenotypes are consistent with their small size during vegetative growth [114]. **(I)** The ∆*trmE* mutant at T1.75 has longer cells; few with polar septa and almost none with σ^F^ activity. At T3.5, membrane engulfment appears aberrant with some unusual membrane invaginations (inset). **(J)** The ∆*gidA* mutant at hour 1.75 and 3.5. ∆*gidA* and ∆*trmE* have similar phenotypes; **(K)** The ∆*ytxG* mutant at hour 1.75, 2.5, and 3.5 of sporulation. The mutant exhibits membrane defects that are most pronounced during engulfment. **(L)** The ∆*rasP* mutant at T1.75 has few cells with polar septa and almost no cells with σ^F^ activity. **(M)** The ∆*ydcC* mutant at hour 5 has small forespores (yellow carets) some with reduced σ^G^ activity. Scale bars are 2 μm.
(PDF)

S8 Fig The *spoIIIL* gene with its σ^F^-responsive promoter complements the *spoIIIL* mutant. **(A)** Representative images of sporulating cells at hour 3.5 in the indicated genetic backgrounds, highlighting complementation of the ∆*spoIIIL* mutant at the level of σ^G^ activity (P_*sspB*_-*cfp*), forespore size, and the formation of heat-resistant spores after sporulation by resuspension. Scale bars are 2 μm. **(B)** Percentage of heat-resistant spores after 24 h in nutrient exhaustion medium (DSM), highlighting the complementation of *spoIIIL*. Histograms from left to right are WT, ∆*spoIIIAH* mutant, ∆*spoIIIL*, ∆*spoIIIAH ∆spoIIIL*, the complemented ∆*spoIIIL* mutant, the complemented ∆*spoIIIAH ∆spoIIIL* double mutant, the complemented ∆*spoIIIL* mutant with an IPTG-inducible *spoIIIL* allele, the complemented *spoIIIAH spoIIIL* double mutant with an IPTG-inducible *spoIIIL* allele. The raw underlying numerical data for S8B Fig can be found in S1 Data.
(PDF)

S9 Fig Cytological phenotype of the Δ*spoIIT* mutant. Large fields of sporulating WT and the ∆*spoIIT* mutant from the experiment in Fig 7B are shown at hour 3 (T3) and hour 5 (T5). Cells were stained with the membrane dye TMA-DPH (shown in red), and express both σ^F^ and σ^E^ activity reporters (green and blue, respectively). Although ~40% of the cells form dormant spores, the majority fail to complete differentiation. Scale bar indicates 1 μm.
(PDF)

S10 Fig Phenotype of the Δ*spoIIT* mutant. **(A)** Quantitation of the ∆*spoIIT* mutant phenotype from the experiment shown in Fig 7B. Bar graph indicates the percentage of sporulating cells at hour 2 with σ^F^ activity, σ^E^ activity, and with two polar septa for each strain. More than 1,000 sporangia were analyzed for each strain. The raw underlying numerical data for S10A Fig can be found in S1 Data. **(B)** Complementation of the Δ*spoIIT* mutant. Representative images of sporulating cells at hour 2 in the indicated genetic backgrounds, highlighting complementation of the ∆*spoIIT* mutant at the level of σ^E^ activity (*P*_*spoIID*_-*mCherry*), loss of the abortive disporic phenotype, and the formation of heat-resistant spores. The complementing allele of *spoIIT* was provided in *trans* under the control of an IPTG-inducible (P_*spank*_) promoter. IPTG was added upon the initiation of sporulation to 500 μM. Scale bar indicates 1 μm. **(C)** Immunoblot analysis of proteolytic processing of pro-σ^E^ in WT, ∆*spoIIT*, ∆*spoIIR*, and ∆*sigE* mutants at the indicated times after the initiation of sporulation. σ^F^ protein levels were analyzed to compare entry into sporulation. **(D)** Immunoblot analysis of SpoIIR-His6 levels in WT and a ∆*spoIIT* mutant at the indicated times after the initiation of sporulation.
(PDF)

S11 Fig *spoIIT* is broadly conserved among endospore-forming bacteria. **(A)** Occurrence of *spoIIT* across the bacterial phylogenetic tree. Red bands indicate the presence of a predicted *spoIIT* ortholog in each taxon. The phylogenetic tree was constructed in PhyloT and was displayed and manually pruned in iTOL. **(B)** The *spoIIT* locus (red) and surrounding chromosomal loci in representative set of endospore-forming bacteria. Also displayed is the corresponding region in *Sporosarcina newyorkensis*, an endospore-forming bacterium that does not contain a predicted *spoIIT* ortholog.
(PDF)

S1 Methods Plasmid and strain construction. (DOCX)

S1 Table Sporulation genes identified at T24 by Tn-seq. (XLSX)

S2 Table Genes that were underrepresented at T5 compared to T0. (XLSX)

S3 Table Regions of the genome in which transposon insertions were overrepresented at T5. (XLSX)

S4 Table List of strains used in this study. (DOCX)

S5 Table List of plasmids used in this study. (DOCX)

S6 Table List of oligonucleotide primers used in this study. (DOCX)

## Abbreviations

CFP: cyan fluorescent protein
CFU: colony forming units
CH: casein hydrolysate
DSM: Difco Sporulation Medium
LB: lysogeny broth
Tn-seq: transposon-sequencing
WT: wild type
YFP: yellow fluorescent protein
