# Supplementary figures and images for: High-Throughput Genetic Screens Identify a Large and Diverse Collection of New Sporulation Genes in Bacillus subtilis
Source: PLoS Biol. 2016 Jan 6;14(1):e1002341. doi: 10.1371/journal.pbio.1002341 (PMC4703394; doi:10.1371/journal.pbio.1002341)

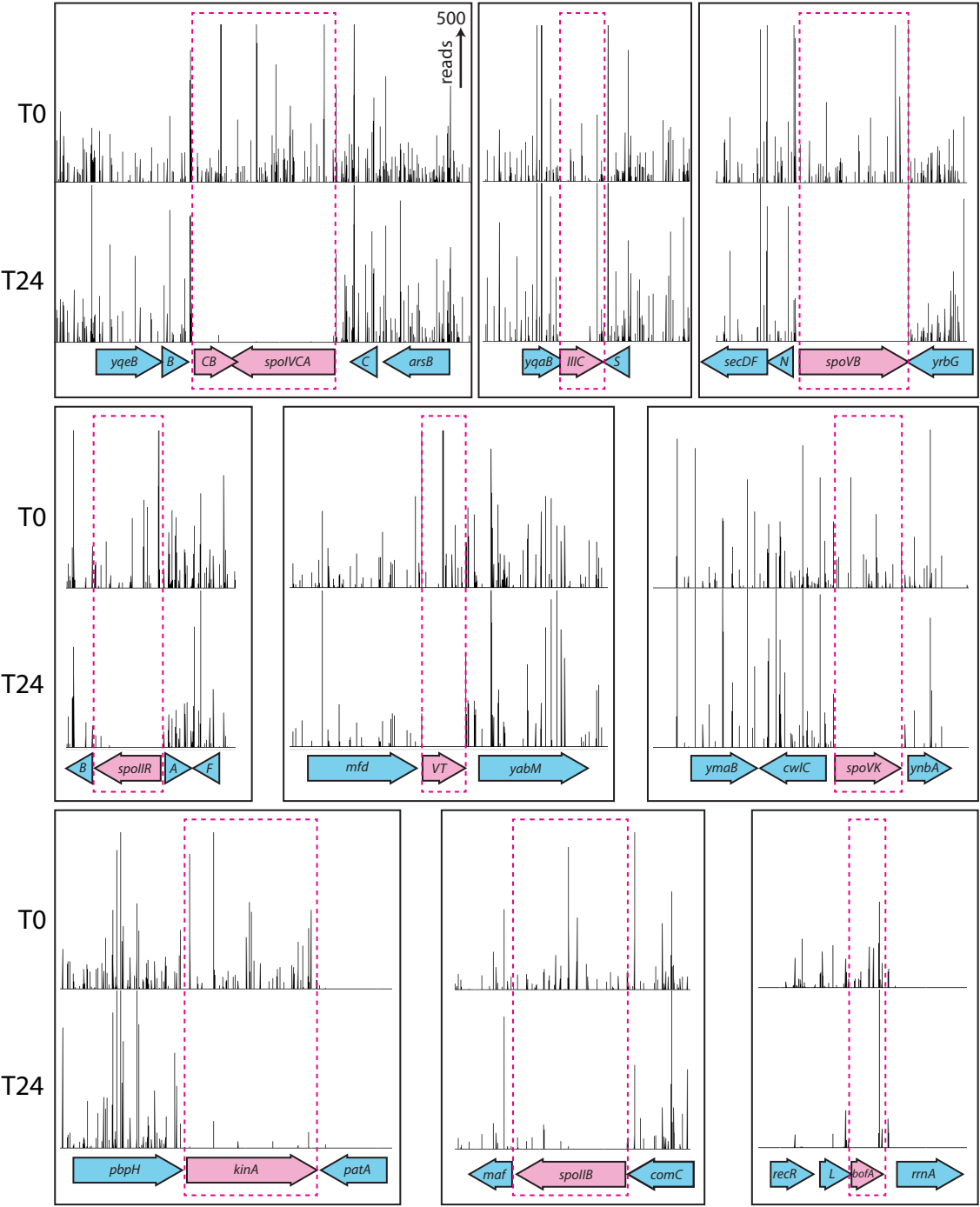

Supplement: S1 Fig — Examples of transposon insertion profiles for ten known and “named” sporulation loci. Transposon insertions at the onset of starvation (T0) and after 24 h (T24) of sporulation are displayed for nine regions of the genome. The height of each vertical line reflects the number of sequencing reads at this position. “VT” refers to spoVT, “CB” to spoIVCB, and “IIIC” to spoIIIC. (PDF) [file pbio.1002341.s002.pdf]

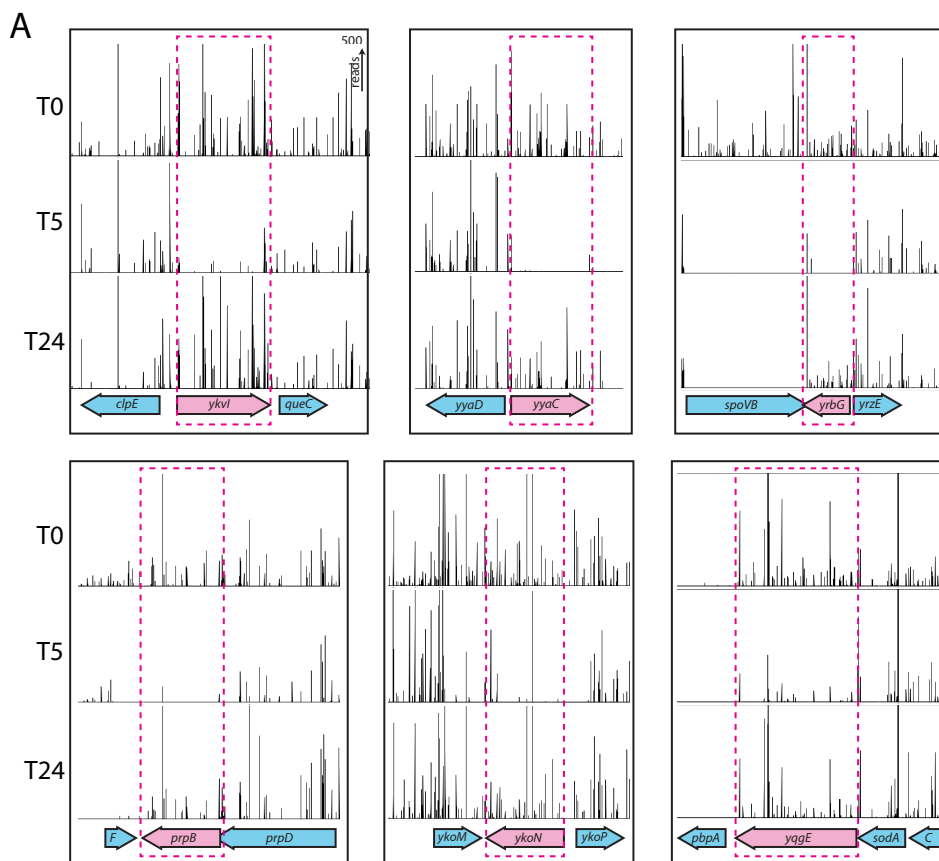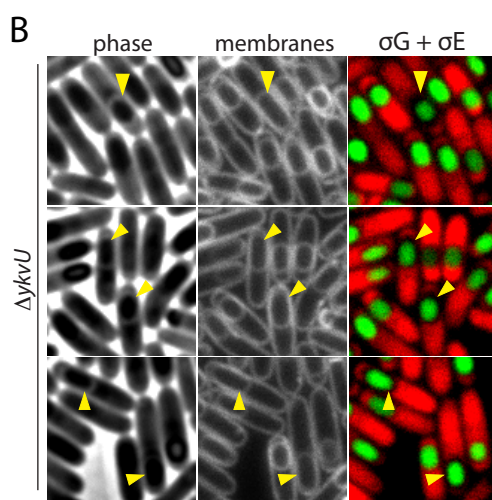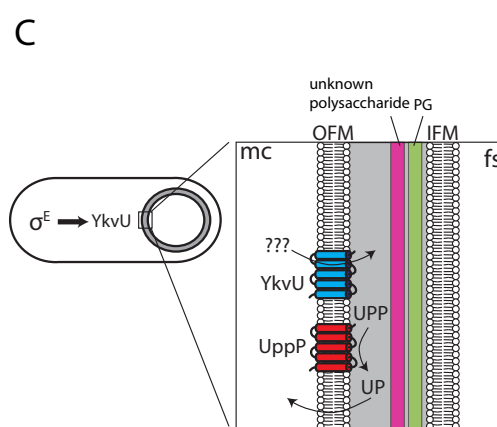

Supplement: S2 Fig — (A) Examples of transposon insertion profiles for six mutants delayed in sporulation. Transposon insertions at the onset of starvation (T0) and after 5 (T5) or 24 (T24) h of sporulation are displayed for six regions of the genome. (B) Additional examples of the ΔykvU mutant phenotype as described in Fig 2B. Phase contrast, membrane staining and overlays of the mother cell (red) and forespore (green) cytoplasmic fluorescent proteins. Yellow carets highlight phase bright rings, the enlarged outer forespore membrane, and the gap between the two compartments. (C) Model for the functions of YkvU and UppP during sporulation. YkvU is a putative flippase produced in the mother cell that could transport lipid-linked precursors into the intermembrane space to synthesize an unidentified polysaccharide. UppP is an undecaprenyl pyrophosphate (UPP) phosphatase potentially involved in the recycling of the undecaprenyl pyrophosphate (UP) lipid carrier. (PDF) [file pbio.1002341.s003.pdf]

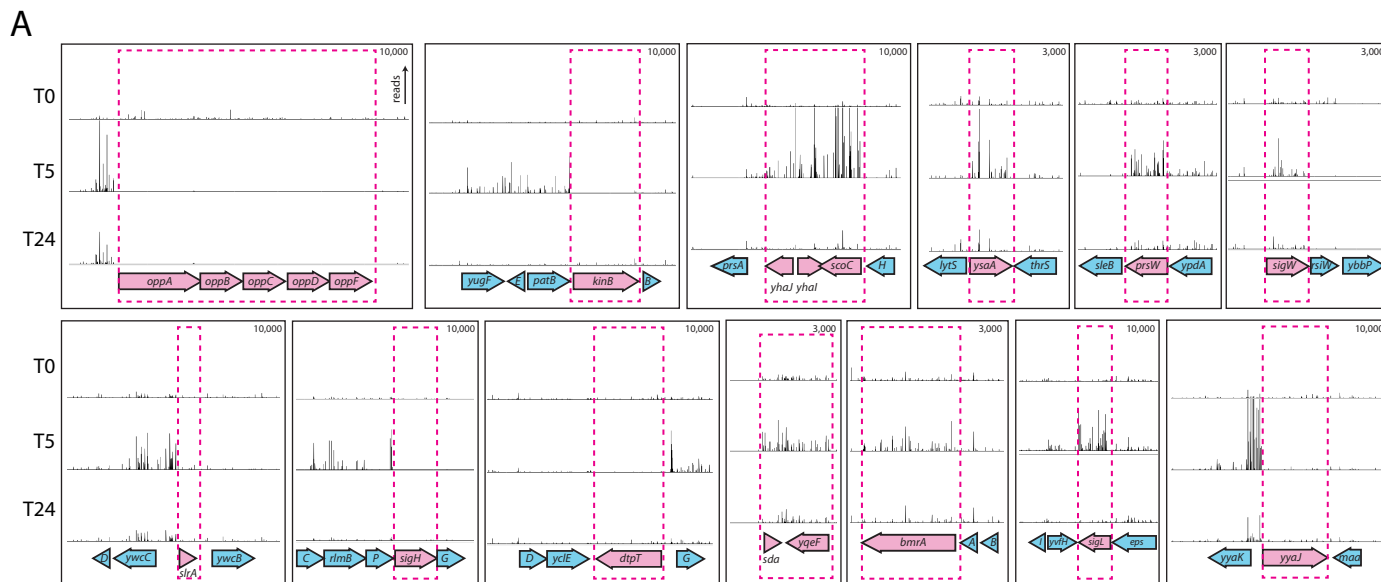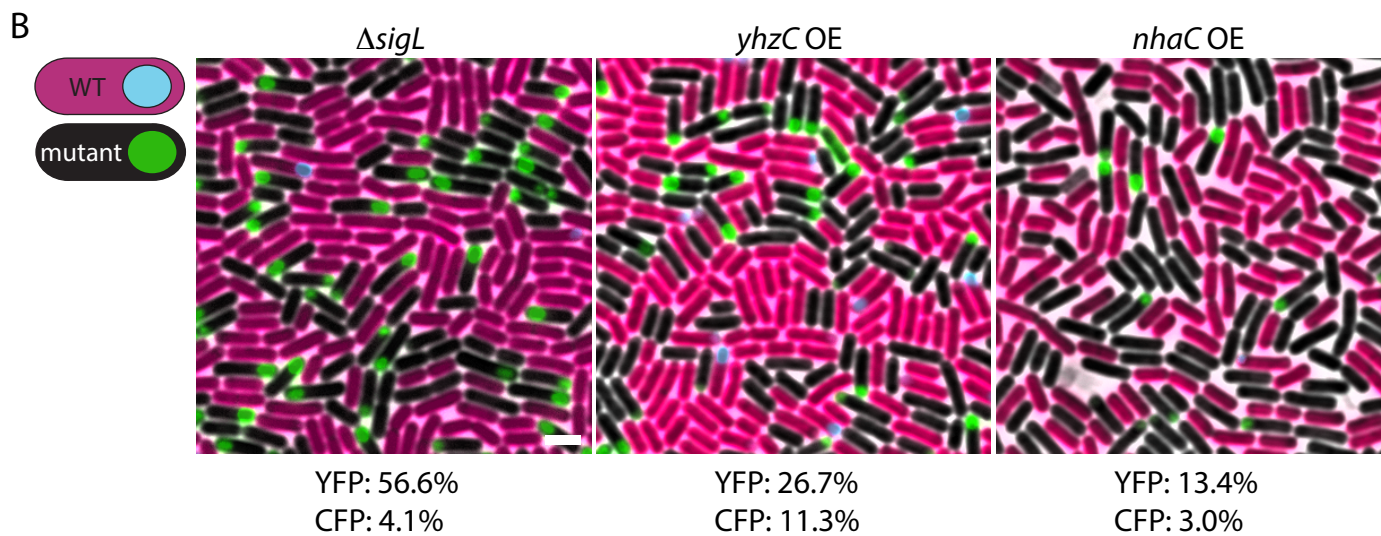

Supplement: S3 Fig — (A) Examples of transposon insertion profiles for 13 regions of the genome at the onset of starvation (T0) and after 5 (T5) or 24 (T24) h of sporulation. The scale of maximum reads is 10,000 (or 3,000 where indicated) to highlight the enrichment of reads at T5. The genes colored pink below the insertion profiles are predicted to be the one impacted by the insertions. For yhaJ, yhaI, scoC, ysaA, prsW, sigW, sda, yqeF, bmrA, and sigL, the insertions are predicted to cause gene inactivation. For opp, kinB, slrA, sigH, dtpT, and yyaJ, the insertions are predicted to cause over-expression. (B) Premature initiation of sporulation in the ΔsigL mutant and strains over-expressing (OE) yhzC and nhaC. All three strains harbor a PspoIIQ-yfp fusion to monitor and early stage of sporulation. Strains were separately mixed 1:1 with wild type (WT) harboring constitutively expressed mCherry and PspoIIQ-cfp. Images are from hour 1 after the onset of starvation. The percentage of sporulating cells (as assayed by σF activity) in the mutant (YFP positive, mCherry negative) and the WT (CFP positive, mCherry positive) are shown for each culture. (PDF) [file pbio.1002341.s004.pdf]

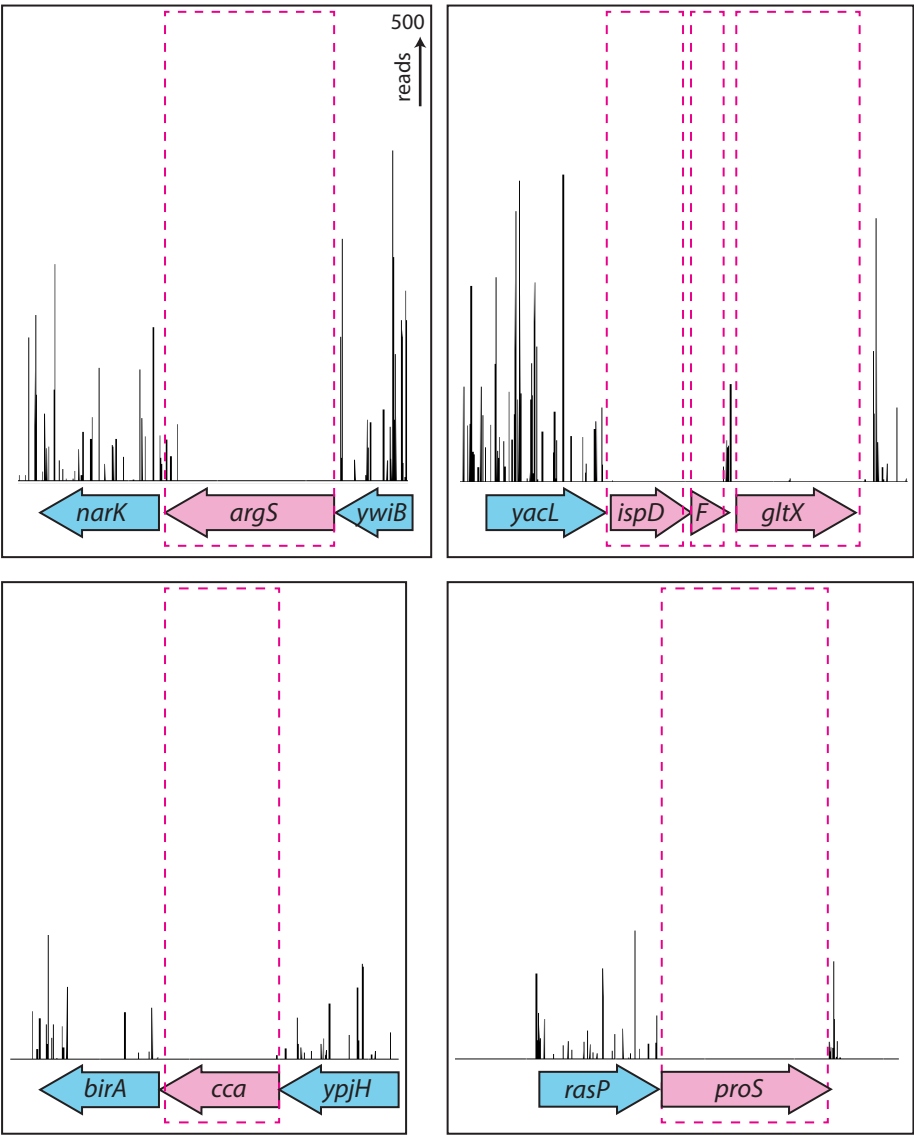

Supplement: S4 Fig — Transposon insertion profiles for four regions of the genome in which an essential gene (pink) resides downstream of a nonessential gene (blue) in an operon. The recovery of insertions in the nonessential blue gene provides evidence that the transposon can promote expression of the downstream essential gene. The profiles depicted are derived from cells harvested at the onset of starvation. (PDF) [file pbio.1002341.s005.pdf]

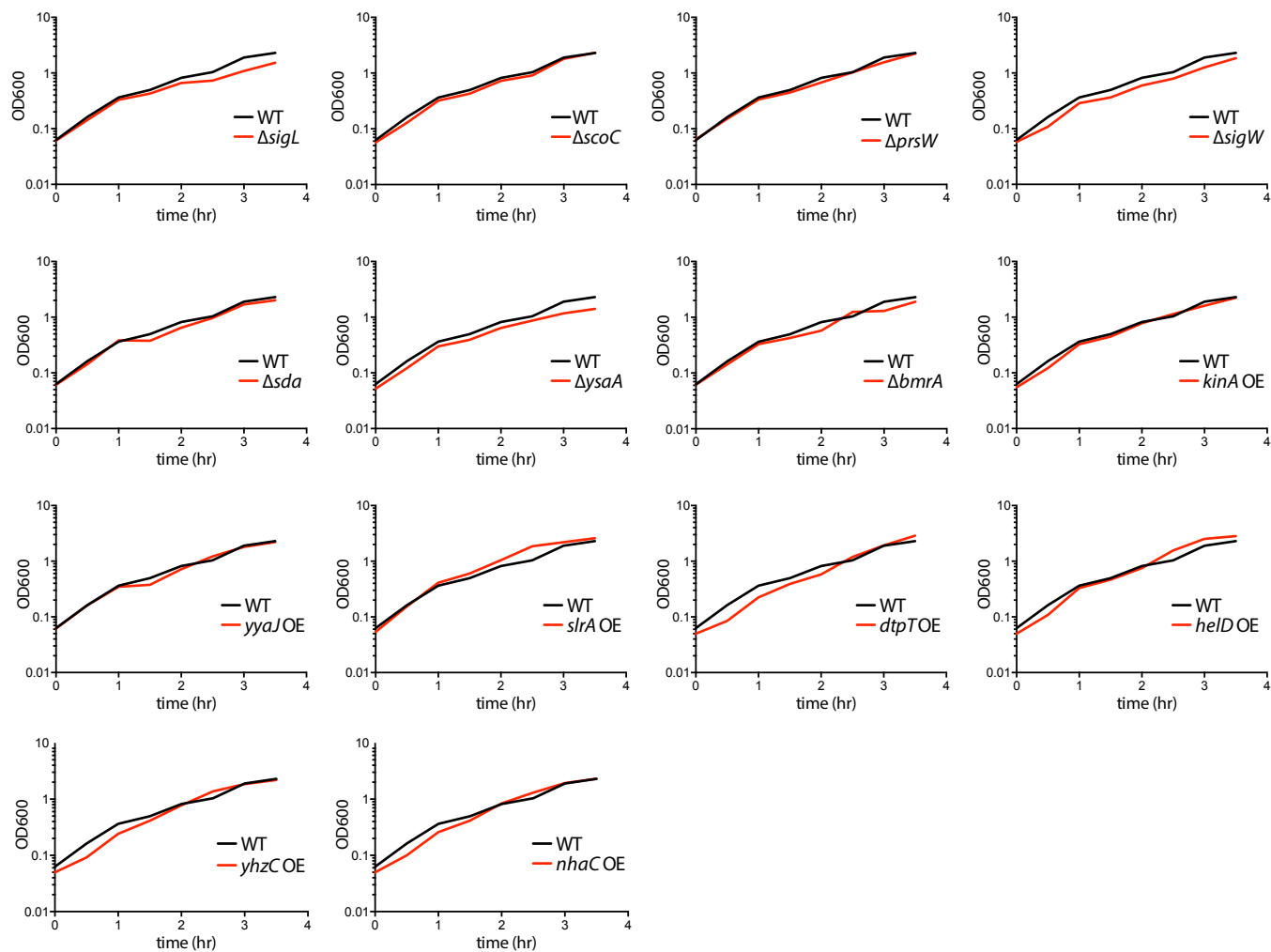

Supplement: S5 Fig — Growth curves of all validated prematurely sporulating mutants. Indicated mutants and over-expressing (OE) strains are shown in comparison to WT. For each growth curve, the WT is depicted in black and the mutant in red. ΔsigL and ΔysaA mutants are the only strains that enter stationary phase earlier than WT. The raw underlying numerical data for S5 Fig can be found in S1 Data. (PDF) [file pbio.1002341.s006.pdf]

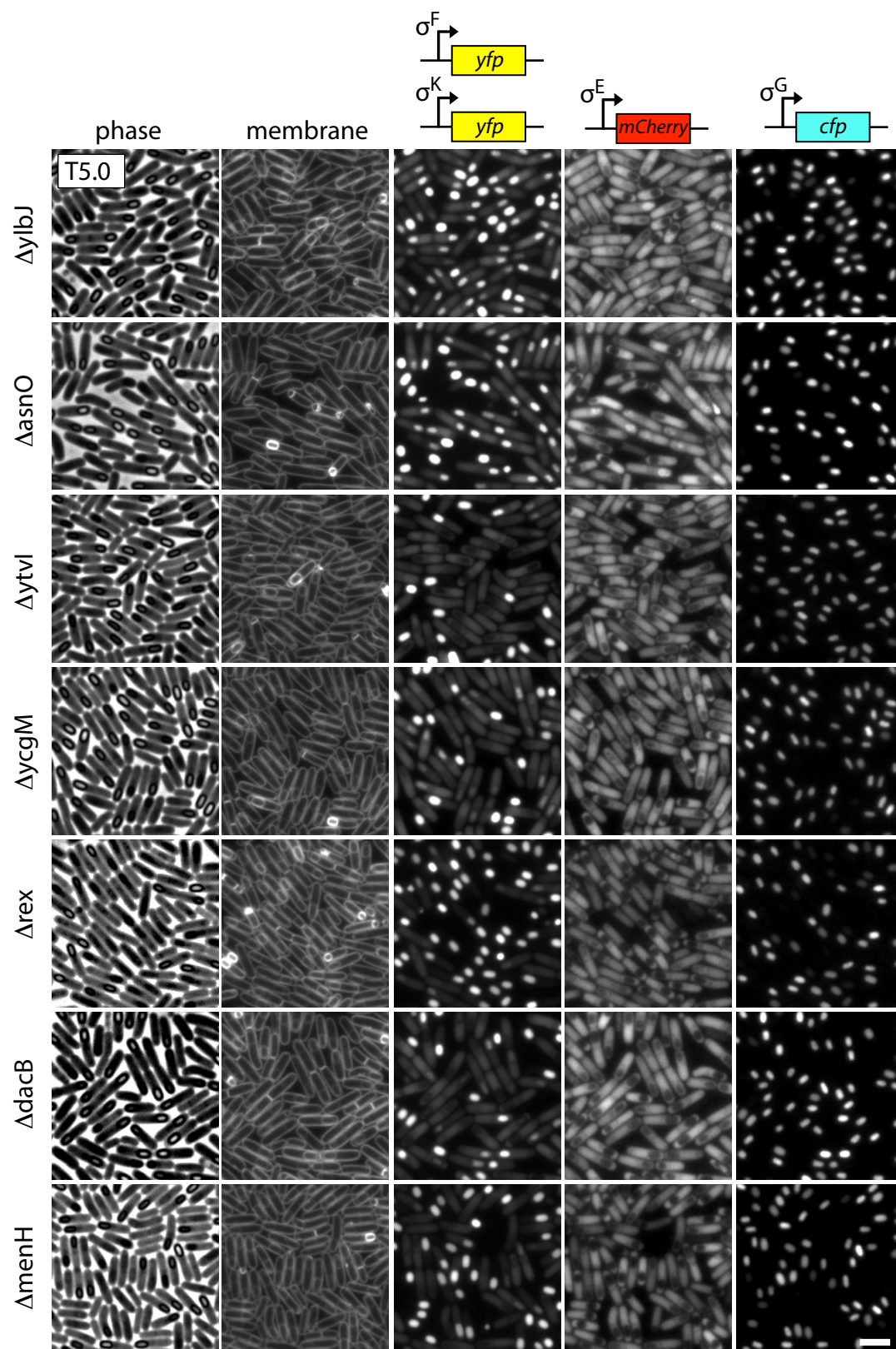

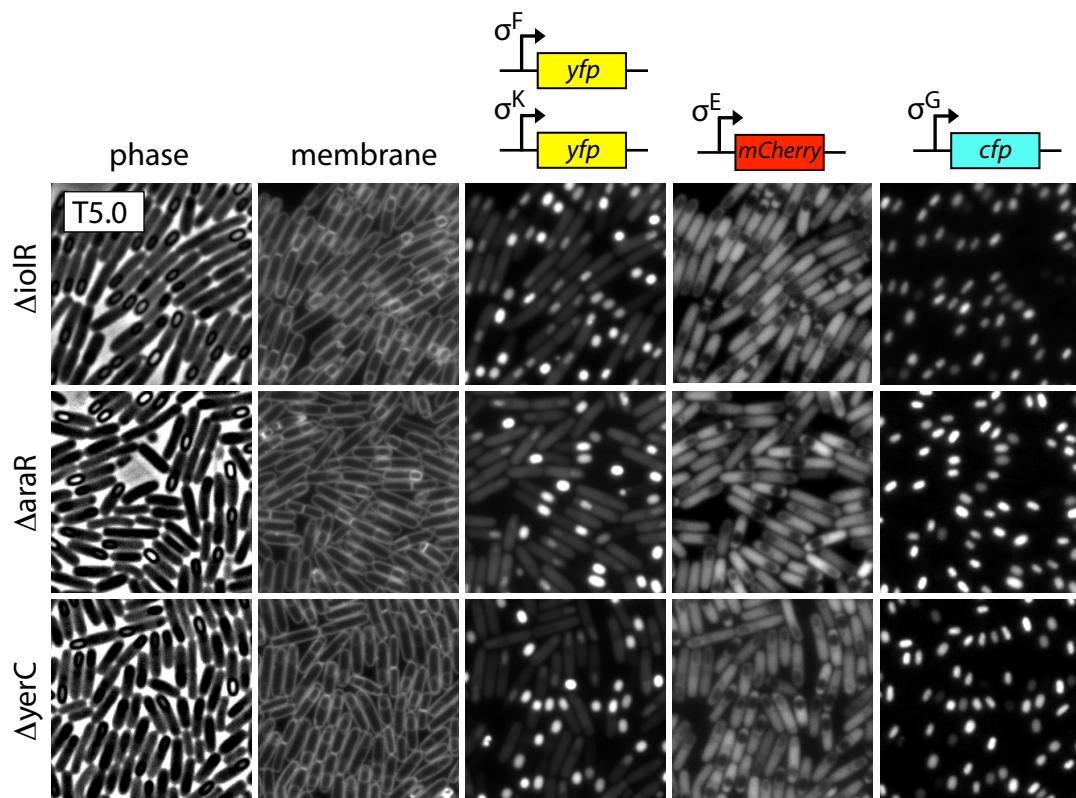

Supplement: S6 Fig — Representative images of ten mutants harboring four transcriptional fusions at hour 5 (T5) of sporulation. Phase contrast, membrane staining and the indicated fluorescent fusions are shown. Scale bars are 2 μm. (PDF) [file pbio.1002341.s007.pdf]

**A**  $\Delta gapB$

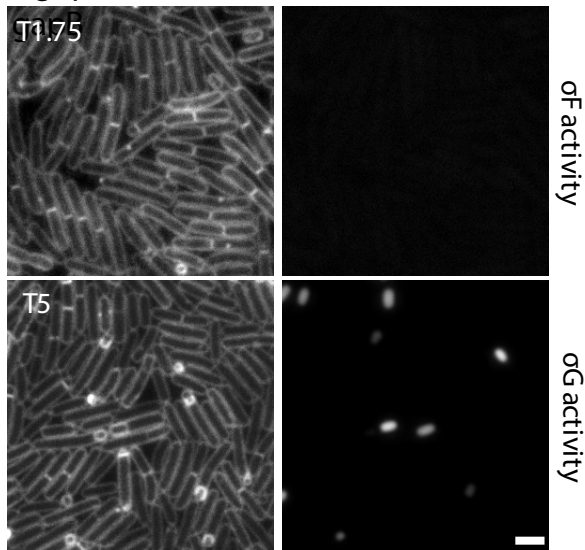

**B**  $\Delta resA$

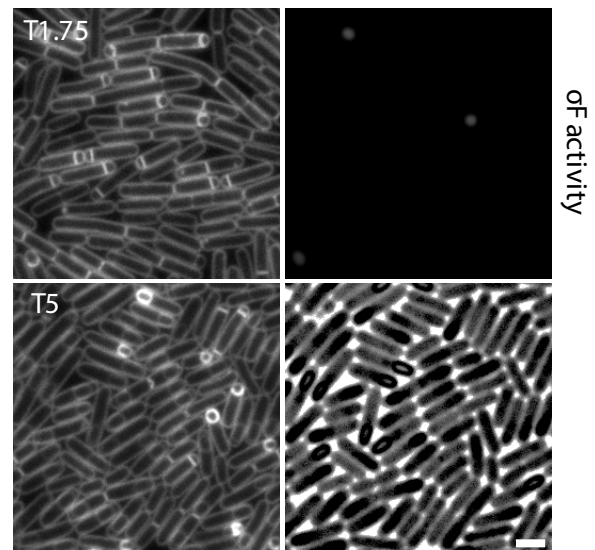

**C**  $\Delta yqhT(\Delta papA)$

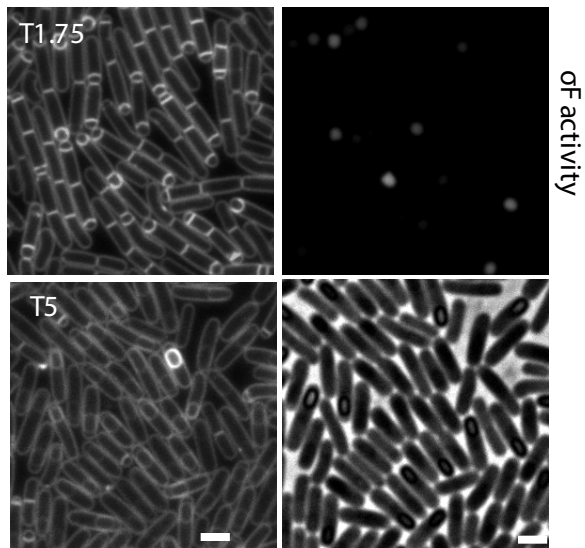

**D**  $\Delta yqfD$

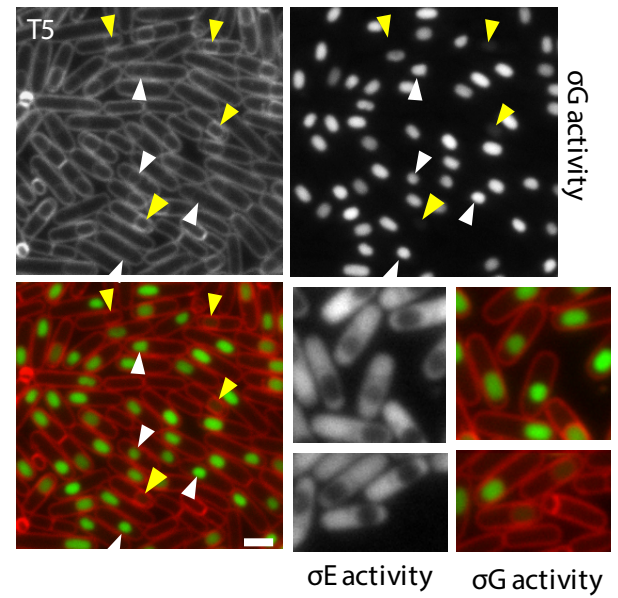

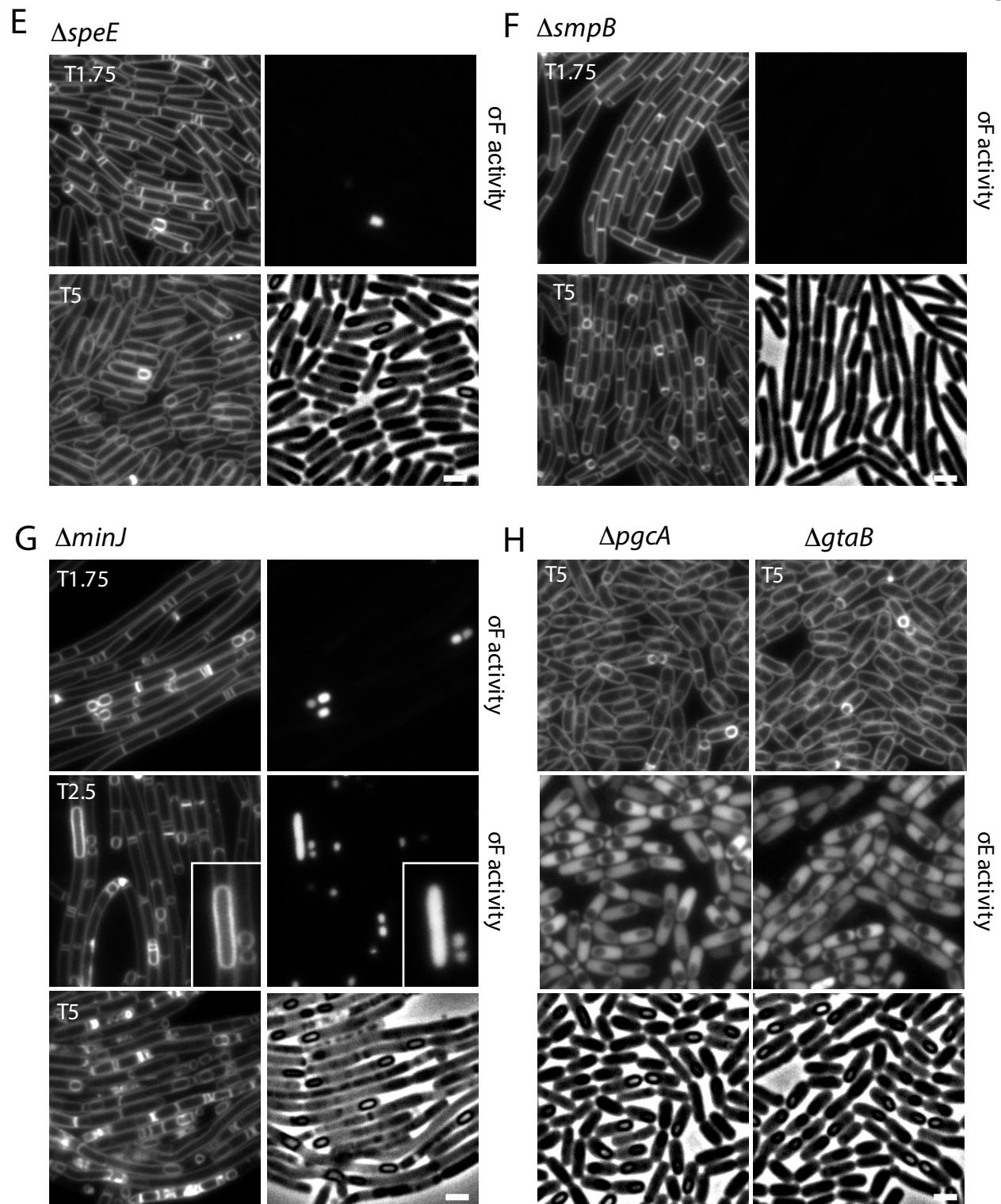

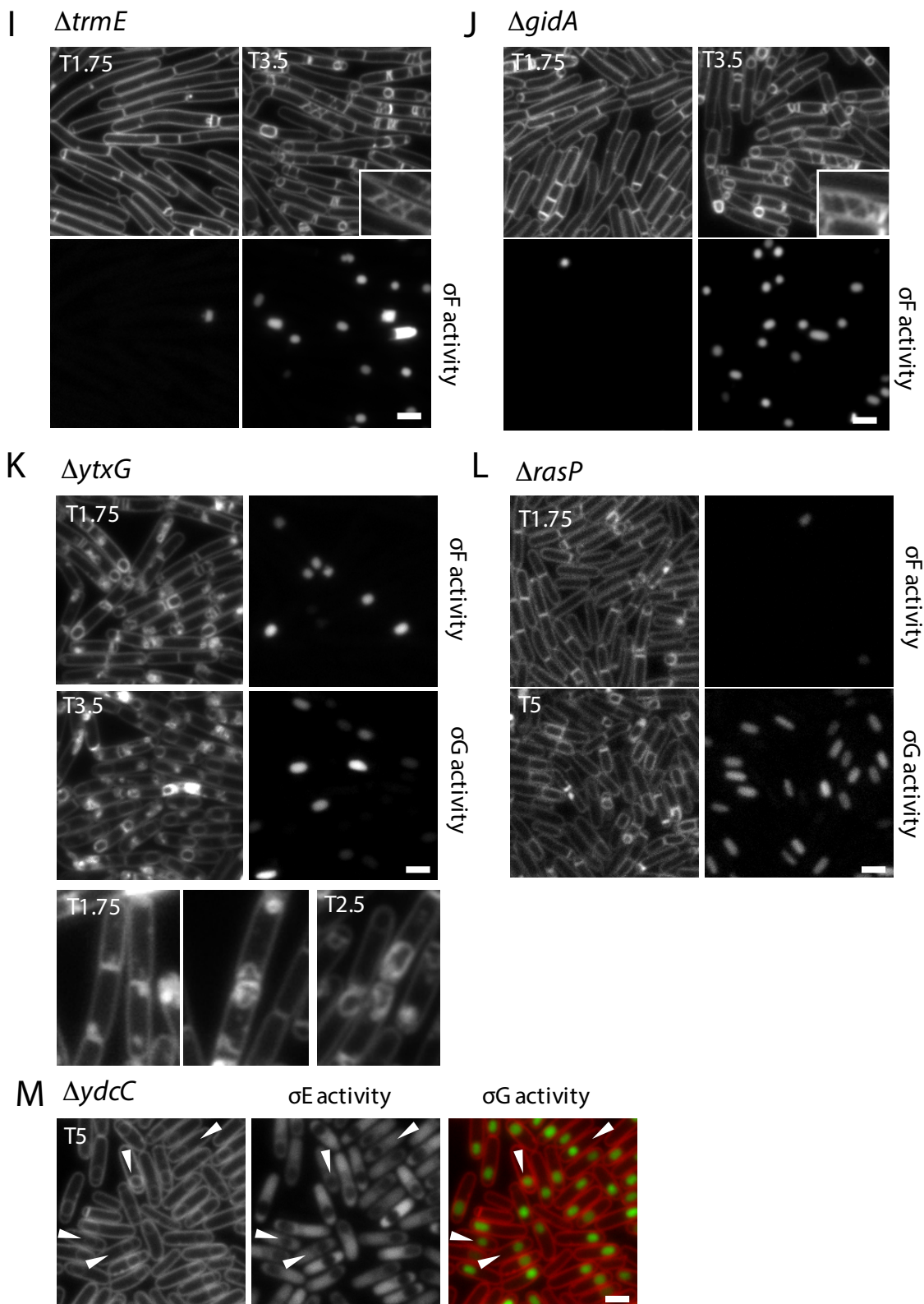

Supplement: S7 Fig — Representative images of 14 mutants harboring four transcriptional fusions at the indicated times after the initiation of sporulation (A) At hour 1.75 (T1.75), the ∆gapB mutant has few polar septa indicative of a delay in entry into sporulation. At hour 5 (T5), very few cells have completed engulfment and most forespores lack detectable σG activity. (B) The ∆resA mutant at T1.75 has few cells with polar septa and a small number with σF activity indicative of an early delay in sporulation. At hour 5 (T5), many cells have completed engulfment but most have not become phase-grey. (C) The ∆yqhT mutant at T1.75 has many cells with polar septa but relatively few with σF activity. At T5, many cells have completed engulfment but most have not become phase-grey. (D) The ∆yqfD mutant at hour 5. A subset of sporulating cells have small forespores (white carets) and reduced σG activity (yellow carets). The two phenotypes are not always correlated in this mutant. Enlarged examples of small forespores and forespores with reduced σG activity are shown on the right. (E) The ∆speE mutant at T1.75 has few cells with polar septa and almost no cells with σF. At T5, many cells have completed engulfment but most have not become phase-grey. (F) The ∆smpB mutant at T1.75 has few cells with polar septa and no σF activity. At T5, most cells have completed engulfment but no forespores are phase-grey. (G) The ∆minJ mutant at T1.75 has few cells with polar septa. At T2.5, some of the engulfed forespores lack σF activity. There are a few large cells with strongly staining membranes that have σF activity (inset). By T5, some spores are phase bright but many sporulating cells have arrested or lysed. (H) The ∆pgcA mutant and the ∆gtaB mutant at hour 5 have relatively normal-sized forespores and small mother cells. These phenotypes are consistent with their small size during vegetative growth [114]. (I) The ∆trmE mutant at T1.75 has longer cells; few with polar septa and almost none with σF activity [file pbio.1002341.s008.pdf]

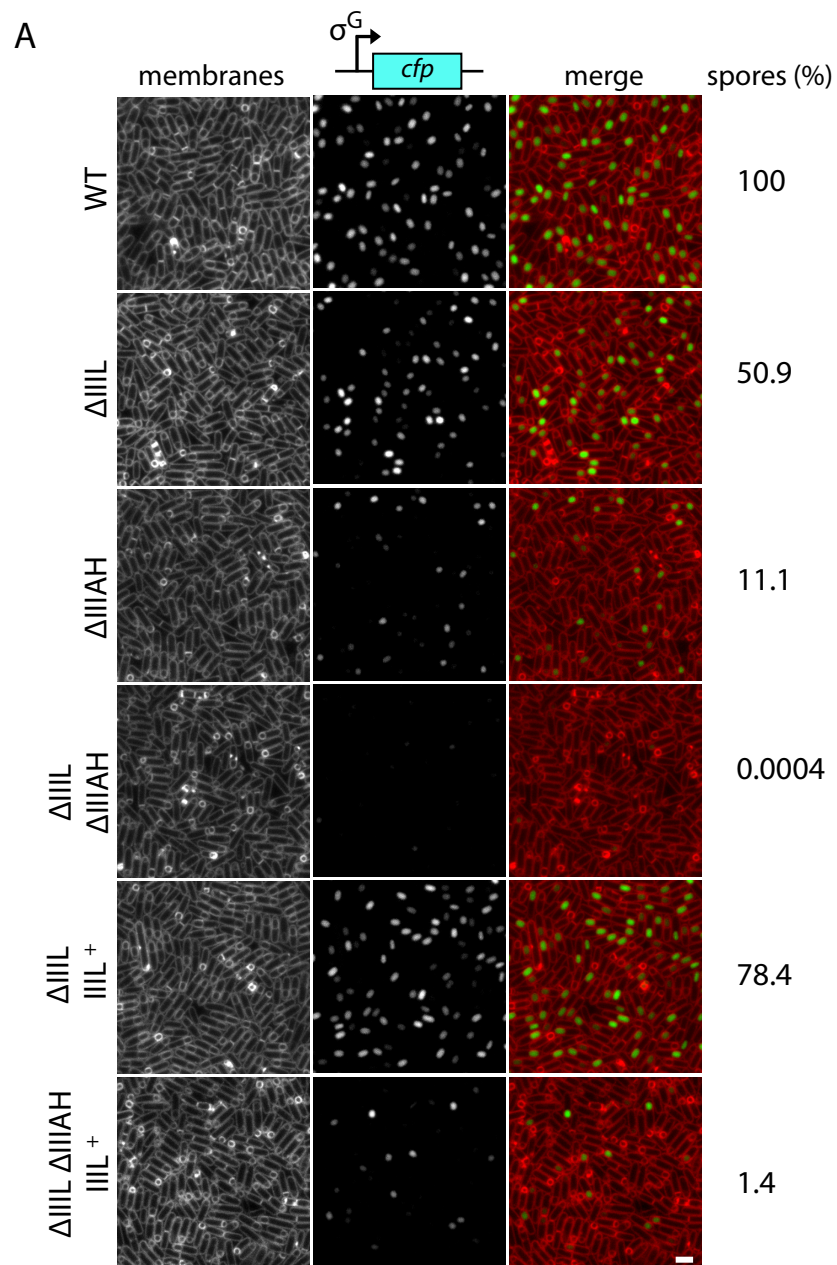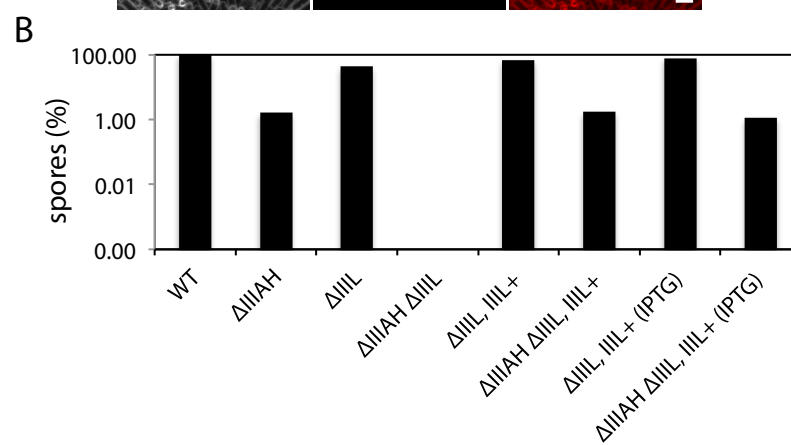

Supplement: S8 Fig — (A) Representative images of sporulating cells at hour 3.5 in the indicated genetic backgrounds, highlighting complementation of the ∆spoIIIL mutant at the level of σG activity (PsspB-cfp), forespore size, and the formation of heat-resistant spores after sporulation by resuspension. Scale bars are 2 μm. (B) Percentage of heat-resistant spores after 24 h in nutrient exhaustion medium (DSM), highlighting the complementation of spoIIIL. Histograms from left to right are WT, ∆spoIIIAH mutant, ∆spoIIIL, ∆spoIIIAH ∆spoIIIL, the complemented ∆spoIIIL mutant, the complemented ∆spoIIIAH ∆spoIIIL double mutant, the complemented ∆spoIIIL mutant with an IPTG-inducible spoIIIL allele, the complemented spoIIIAH spoIIIL double mutant with an IPTG-inducible spoIIIL allele. The raw underlying numerical data for S8B Fig can be found in S1 Data. (PDF) [file pbio.1002341.s009.pdf]

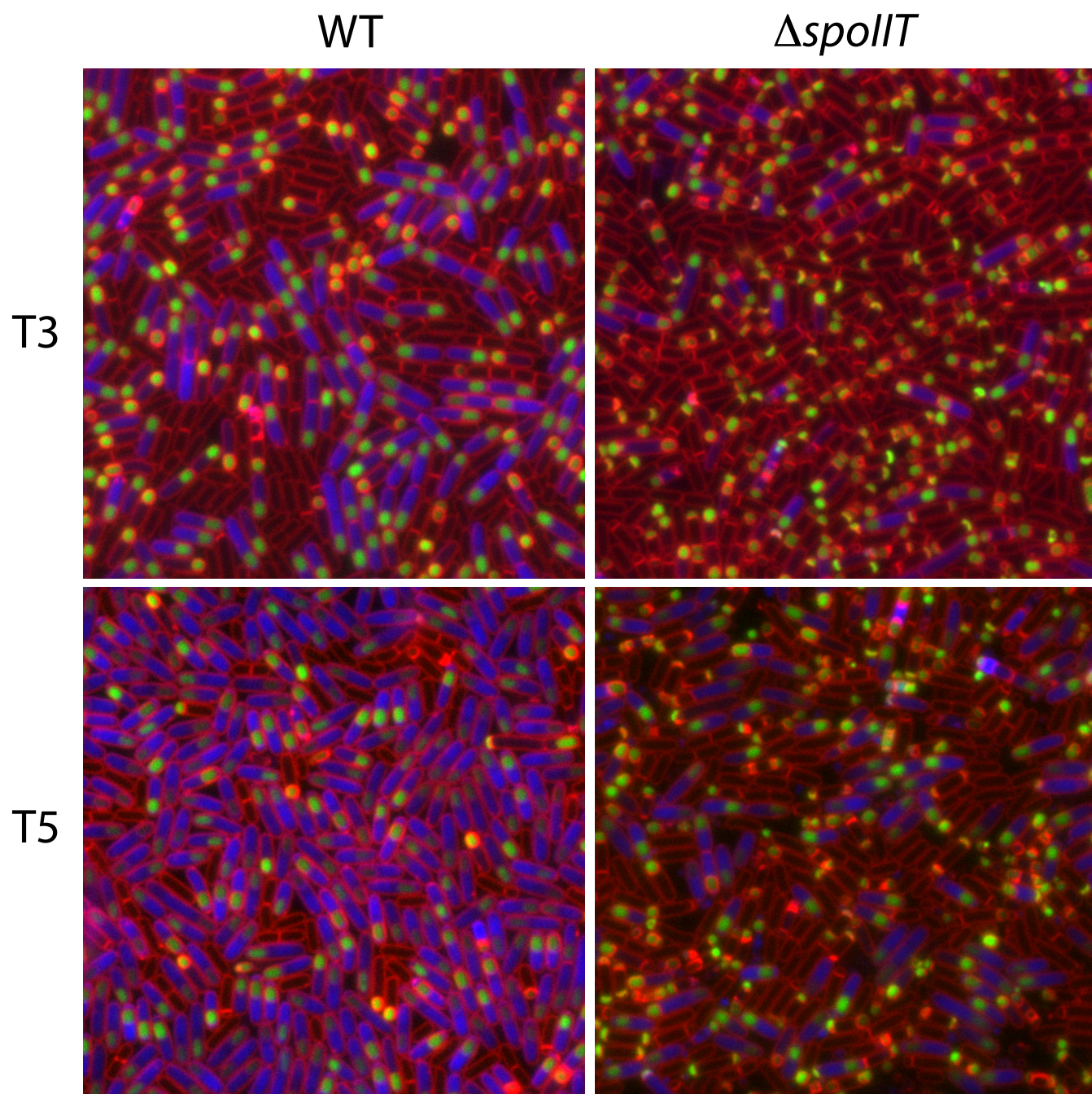

Supplement: S9 Fig — Large fields of sporulating WT and the ∆spoIIT mutant from the experiment in Fig 7B are shown at hour 3 (T3) and hour 5 (T5). Cells were stained with the membrane dye TMA-DPH (shown in red), and express both σF and σE activity reporters (green and blue, respectively). Although ~40% of the cells form dormant spores, the majority fail to complete differentiation. Scale bar indicates 1 μm. (PDF) [file pbio.1002341.s010.pdf]

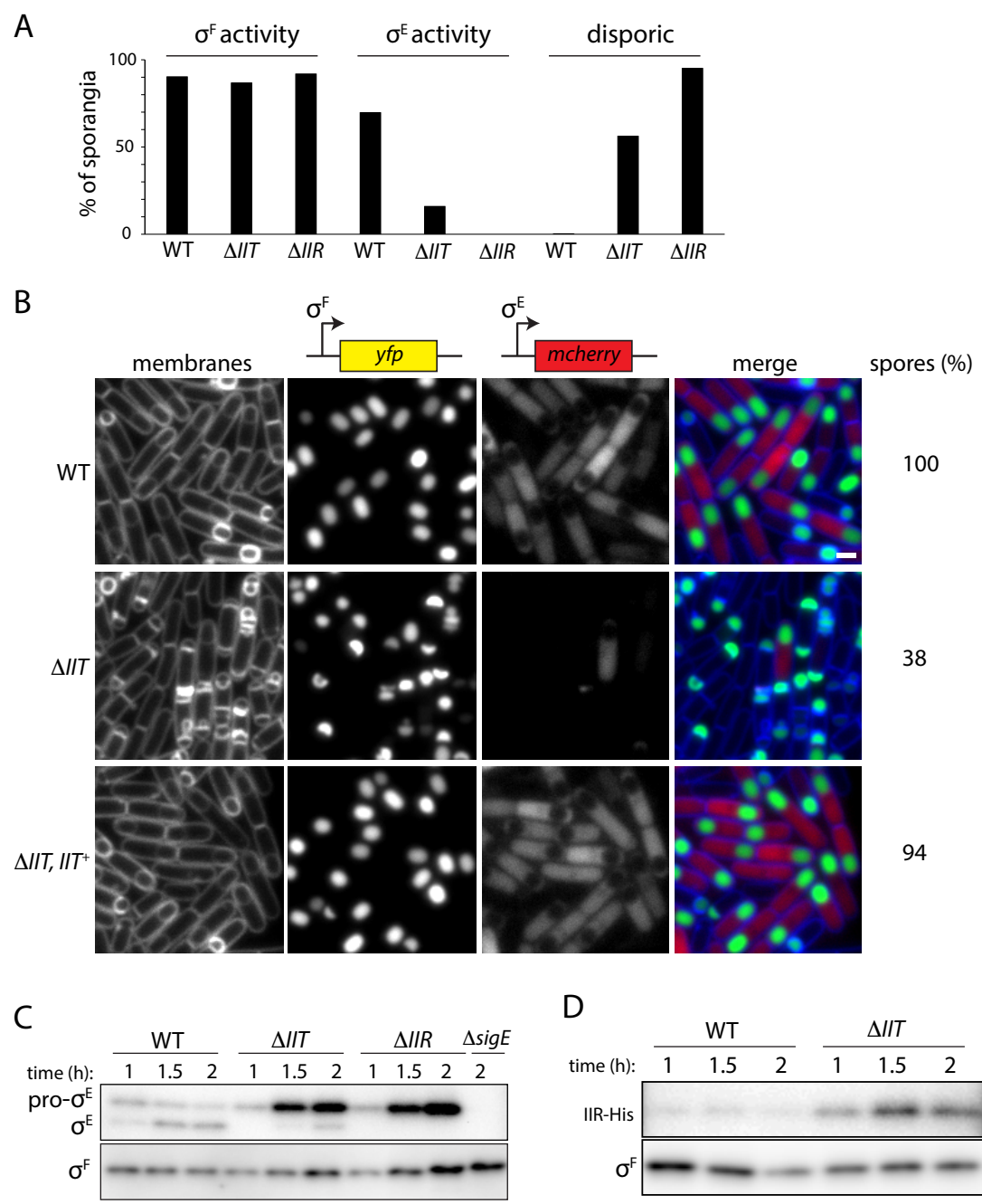

Supplement: S10 Fig — (A) Quantitation of the ∆spoIIT mutant phenotype from the experiment shown in Fig 7B. Bar graph indicates the percentage of sporulating cells at hour 2 with σF activity, σE activity, and with two polar septa for each strain. More than 1,000 sporangia were analyzed for each strain. The raw underlying numerical data for S10A Fig can be found in S1 Data. (B) Complementation of the ΔspoIIT mutant. Representative images of sporulating cells at hour 2 in the indicated genetic backgrounds, highlighting complementation of the ∆spoIIT mutant at the level of σE activity (PspoIID-mCherry), loss of the abortive disporic phenotype, and the formation of heat-resistant spores. The complementing allele of spoIIT was provided in trans under the control of an IPTG-inducible (Pspank) promoter. IPTG was added upon the initiation of sporulation to 500 μM. Scale bar indicates 1 μm. (C) Immunoblot analysis of proteolytic processing of pro-σE in WT, ∆spoIIT, ∆spoIIR, and ∆sigE mutants at the indicated times after the initiation of sporulation. σF protein levels were analyzed to compare entry into sporulation. (D) Immunoblot analysis of SpoIIR-His6 levels in WT and a ∆spoIIT mutant at the indicated times after the initiation of sporulation. (PDF) [file pbio.1002341.s011.pdf]

A

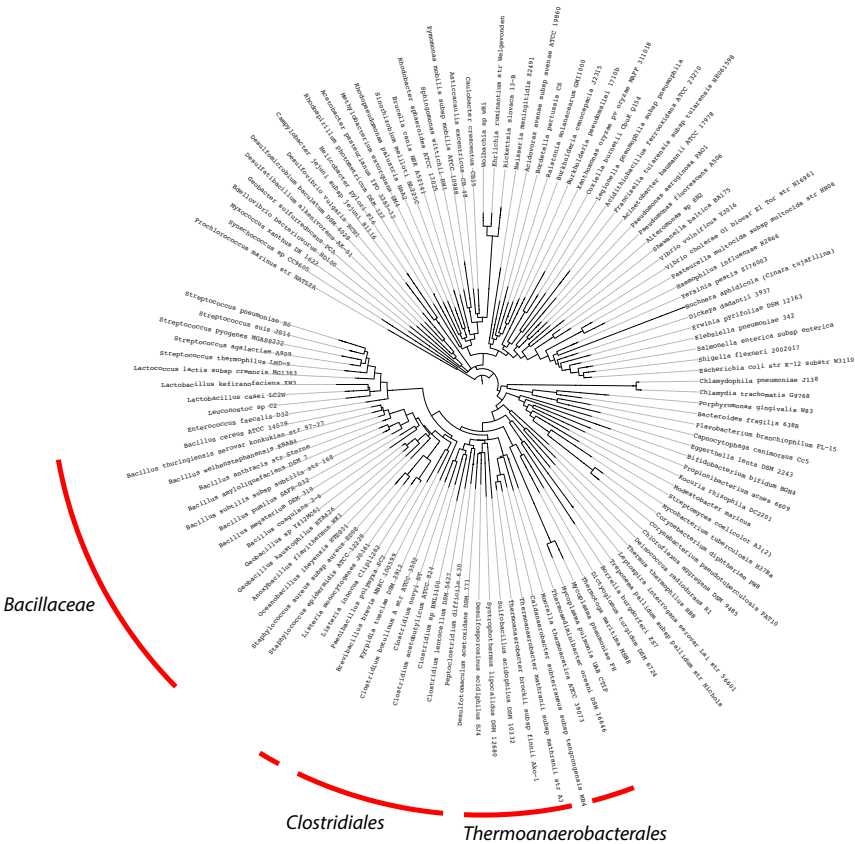

B

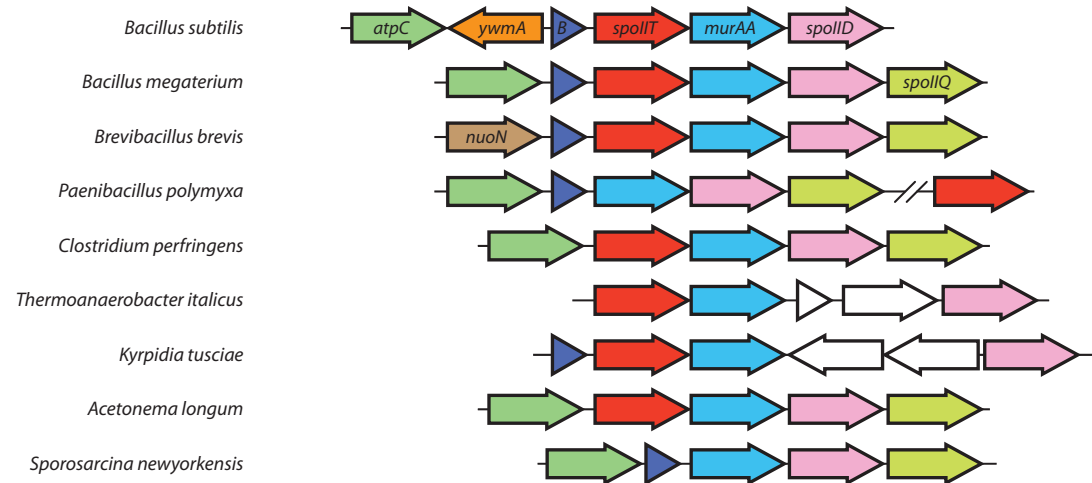

Supplement: S11 Fig — (A) Occurrence of spoIIT across the bacterial phylogenetic tree. Red bands indicate the presence of a predicted spoIIT ortholog in each taxon. The phylogenetic tree was constructed in PhyloT and was displayed and manually pruned in iTOL. (B) The spoIIT locus (red) and surrounding chromosomal loci in representative set of endospore-forming bacteria. Also displayed is the corresponding region in Sporosarcina newyorkensis, an endospore-forming bacterium that does not contain a predicted spoIIT ortholog. (PDF) [file pbio.1002341.s012.pdf]
