# Supplementary material for: High-Throughput Genetic Screens Identify a Large and Diverse Collection of New Sporulation Genes in Bacillus subtilis
Source: PLoS Biol. 2016 Jan 6;14(1):e1002341. doi: 10.1371/journal.pbio.1002341 (PMC4703394; doi:10.1371/journal.pbio.1002341)
Supplement: S1 Methods — (DOCX) [file pbio.1002341.s013.docx]

**Supplemental methods:**

**Strain construction:**

Strain BAM862 [Δ*spoIIT(63aa)::lox72*] was constructed via isothermal assembly and direct transformation into *B. subtilis* of three fragments: *spoIIT* upstream PCR product (oligonucleotide primers oAM433 and oAM434), *spoIIT* downstream PCR product (oligonucleotide primers oAM435 and oAM436), and a loxP-flanked erythromycin resistance cassette from pDR242 which allows transcriptional read through of downstream genes (amplified using oligonucleotide primers oDR1043 and oDR1044). The erm cassette was removed using the Cre/lox system.

Strain BAM892 [*spoIIR-his6* *spec*] was constructed via isothermal assembly and direct transformation into *B. subtilis* of three fragments: *spoIIR-his6* upstream PCR product (oligonucleotide primers oAM468 and oAM469), *spoIIR* downstream PCR product (oligonucleotide primers oAM470 and oAM471), and a spectinomycin resistance cassette (amplified from pWX466 using oligonucleotide primers oJM28 and oJM29).

Strains BAM1052 [*magellan6x-dtpT (spec)*] and BAM1053 [*magellan6x-helD (spec)*] were constructed via isothermal assembly and direct transformation into *B. subtilis* of three fragments: upstream PCR product (oligonucleotide primers oAM505 and oAM506 for *dtpT*, oAM509 and oAM510 for *helD),* downstream PCR product (oligonucleotide primers oAM507 and oAM508 for *dtpT*, oAM511 and oAM512 for *helD*), and the *magellan6x* transposon (oligonucleotide primers oAM497 and oAM498).

Antibiotic cassette removal was carried out using the Cre/lox system (previously described in Meeske et al. 2015). Briefly, *B. subtilis* strains carrying a loxP-flanked antibiotic resistance cassette were transformed with pDR244 (temperature-sensitive plasmid with constitutively expressed Cre recombinase marked with a spectinomycin resistance cassette). Transformants were selected on LB supplemented with 100 μg/ml spectinomycin at 30°C, a permissive temperature for pDR244 replication. Transformants were then streaked on LB without antibiotic and incubated at 42°C, a non-permissive temperature for plasmid replication. Single colonies were then re-streaked on LB, LB(spec), and LB containing the antibiotic for which the removed cassette provided resistance and incubated 37°C. Strains that grew on LB, but not the two antibiotics had lost pDR244 and the resistance cassette. Markerless deletions were confirmed by PCR with oligonucleotide primers flanking the deletion.

**Plasmid construction:**

**pAM139** [*lacA::PgerE-yfp (tet)*] was generated by subcloning of the EcoRI-BamHI fragment containing *PgerE-yfp* from pNC165 (Campo and Rudner, unpublished) into the pNC18 *lacA::tet* integration.

**pCR100** [*amyE::PspoIID-mCherry (spec)*] was generated in a two-way ligation with a *Hind*III-*Bam*HI insert containing mCherry from pER099 (Riley and Rudner, unpublished) and pKM022 [*amyE::PspoIID-yfp (spec)*] digested with *Hind*III-*Bam*HI. pKM022 is an ectopic integration vector for double-crossover insertions into the *amyE* gene.

**pCR218** [*yhdG::PyqzE-yqzE (tet)*] was generated in a two-way ligation with an *Eco*RI-*Bam*HI PCR product containing the *yqzE* gene and its promoter region (oligonucleotide primers oCR467 and oCR475 and PY79 genomic DNA) and pBB281 [*yhdG::tet*] cut with *Eco*RI and *Bam*HI. pBB281 is an ectopic integration vector for double-crossover insertions into the *yhdG* gene.

**pCR219** [*yhdG::Pspank-optRBS-yqzE (lacI) (phleo)*] was generated in a two-way ligation with an *Hind*III-*Nhe*I PCR product containing the *yqzE* gene (oligonucleotide primers oCR476 and oCR477 and PY79 genomic DNA) and pBR114 [*yhdG::Pspank (phleo)*] cut with *Hind*III and *Nhe*I. pRB114 is an ectopic integration vector, with an IPTG-inducible promoter, used for double-crossover insertions into the *yhdG* gene.

**pCR220** [*amyE::PyqzE-optRBS-yfp (spec)*] was generated in a three-way ligation with an *Eco*RI-*Hind*III PCR product containing the *yqzE* promoter region (oligonucleotide primers oCR467 and oCR482 and PY79 genomic DNA) and a *Hind*III-*Bam*HI fragment containing the *yfp* gene (oligonucleotide primers oCR783 and oDR078 and pKM162 [*amyE::PspoIIQ-yfp (spec)*] DNA) and plasmid pKM162 [*amyE::PspoIIQ-yfp (spec)*] cut with *Eco*RI and *Bam*HI. pKM162 is an ectopic integration vector for double-crossover insertions into the *amyE* gene.

**pAM161** [*amyE::Phyperspank-yyaJ (spec)*] was generated in a two-way ligation with an *Hind*III-*Nhe*I PCR product containing the *yyaJ* gene (oligonucleotide primers oAM428 and oAM429 and 168 genomic DNA) and pDR111 [*amyE::Phyperspank (spec)*] cut with *Hind*III and *Nhe*I. pDR111 is an ectopic integration vector, with the strong IPTG-inducible Phyperspank promoter, used for double-crossover insertions into the *amyE* gene.

**pAM162** [*amyE::Phyperspank-slrA (spec)*] was generated in a two-way ligation with an *Hind*III-*Nhe*I PCR product containing the *slrA* gene (oligonucleotide primers oAM430 and oAM431 and 168 genomic DNA) and pDR111 [*amyE::Phyperspank (spec)*] cut with *Hind*III and *Nhe*I. pDR111 is an ectopic integration vector, with the strong IPTG-inducible Phyperspank promoter, used for double-crossover insertions into the *amyE* gene.

**pAM165** [*ycgO::Pspank-spoIIT (cat)*] was generated in a two-way ligation with an *Xma*I-*Nhe*I PCR product containing the *spoIIT* gene (oligonucleotide primers oAM437 and oAM438 and 168 genomic DNA) and pAM11 [*ycgO::Pspank (cat)*] cut with *Xma*I and *Nhe*I. pAM11 is an ectopic integration vector, with the IPTG-inducible Pspank promoter, used for double-crossover insertions into the *ycgO* gene.

**pAM175** [*amyE::PspoIIT(small)-yfp (cat)*] was generated in a two-way ligation with an *Eco*RI-*Hind*III PCR product containing 360 bp of sequence preceding the *spoIIT* start codon (oligonucleotide primers oAM459 and oAM460 and 168 genomic DNA) and pNS8 [*amyE::PspoIIQ-yfp (cat)*] cut with *Eco*RI and *Hind*III. pNS8 is an ectopic integration vector used for double-crossover insertions into the *amyE* gene.

**pAM176** [*amyE::PspoIIT(large)-yfp (cat)*] was generated in a two-way ligation with an *Eco*RI-*Hind*III PCR product containing 774 bp of sequence preceding the *spoIIT* start codon (oligonucleotide primers oAM461 and oAM460 and 168 genomic DNA) and pNS8 [*amyE::PspoIIQ-yfp (cat)*] cut with *Eco*RI and *Hind*III. pNS8 is an ectopic integration vector used for double-crossover insertions into the *amyE* gene.

**pAM193** [*amyE::Phyperspank-optRBS-yhzC (spec)*] was generated by isothermal assembly of a PCR product containing the *yhzC* gene (oligonucleotide primers oAM489 and oAM490 and 168 genomic DNA) and pDR111 [*amyE::Phyperspank (spec)*] cut with *Hind*III and *Nhe*I. pDR111 is an ectopic integration vector, with the strong IPTG-inducible Phyperspank promoter, used for double-crossover insertions into the *amyE* gene.

**pAM195** [*amyE::Phyperspank-optRBS-nhaC (spec)*] was generated by isothermal assembly of a PCR product containing the *nhaC* gene (oligonucleotide primers oAM493 and oAM494 and 168 genomic DNA) and pDR111 [*amyE::Phyperspank (spec)*] cut with *Hind*III and *Nhe*I. pDR111 is an ectopic integration vector, with the strong IPTG-inducible Phyperspank promoter, used for double-crossover insertions into the *amyE* gene.
