# Supplementary material for: High-Throughput Genetic Screens Identify a Large and Diverse Collection of New Sporulation Genes in Bacillus subtilis
Source: PLoS Biol. 2016 Jan 6;14(1):e1002341. doi: 10.1371/journal.pbio.1002341 (PMC4703394; doi:10.1371/journal.pbio.1002341)
Supplement: S4 Table — (DOCX) [file pbio.1002341.s017.docx]

**Table S5. List of strains used in this study.**

| **Strain** | **Genotype** | **Source** |
| --- | --- | --- |
| **BDR2413 (168)** | *trpC2* | Zeigler et al. 2008 |
| **BDR11 (PY79)** | Prototrophic wild-type | Youngman et al. 1983 |
| **BTD1609*** | *yycR::PsspB-rbsopt-cfp (phleo)* | Doan et al., 2009 |
| **BCR43*** | *spoIIIAH::spec* | Rodrigues et al., 2013 |
| **BCR176*** | *yycR::PsspB-rbsopt-cfp (phleo), spoIIIAH::erm* | Rodrigues et al., 2013 |
| **BCR826*** | *yycR::PsspB-rbsopt-cfp (phleo), spoIIIA::kan* | This work |
| **BCR1071** | *yycR::PsspB-rbsopt-cfp (phleo), amyE::PspoIID-mCherry(spec), pelB::PspoIIQ-yfp (kan), lacA::PgerE-yfp (tet)* | This work |
| **BCR1191*** | *yycR::PsspB-rbsopt-cfp (phleo), spoIIIL::erm* | This work |
| **BCR1201*** | *yycR::PsspB-rbsopt-cfp (phleo), spoIIIL::erm, spoIIIAH::spec* | This work |
| **BCR1236*** | *spoIIIL::erm* | This work |
| **BCR1244*** | *spoIIIL::erm, spoIIIAH::spec* | This work |
| **BCR1249*** | *yycR::PsspB-rbsopt-cfp (phleo), spoIIIL::erm, yhdG::PspoIIIL-spoIIIL (tet)* | This work |
| **BCR1250*** | *yycR::PsspB-rbsopt-cfp (phleo), spoIIIL::erm, spoIIIAH::spec, yhdG::PspoIIIL-spoIIIL (tet)* | This work |
| **BCR1251*** | *spoIIIL::erm, yhdG::PspoIIIL-spoIIIL (tet)* | This work |
| **BCR1252*** | *spoIIIL::erm, spoIIIAH::spec, yhdG::PspoIIIL-spoIIIL (tet)* | This work |
| **BCR1254*** | *spoIIIL::erm, yhdG::Pspank-spoIIIL (phleo)* | This work |
| **BCR1256*** | *spoIIIL::erm, spoIIIAH::spec, yhdG::Pspank-spoIIIL (phleo)* | This work |
| **BCR1264*** | *amyE::PspoIIIL-optRBS-YFP (spec)* | This work |
| **BCR1266*** | *amyE::PspoIIIL-optRBS-YFP (spec), spoIIAC::kan* | This work |
| **BAM716** | *pgcA::erm, yycR::PsspB-rbsopt-cfp (phleo), amyE::PspoIID-mCherry(spec), pelB::PspoIIQ-yfp (kan), lacA::PgerE-yfp (tet)* | This work |
| **BAM717** | *ybbP::erm, yycR::PsspB-rbsopt-cfp (phleo), amyE::PspoIID-mCherry(spec), pelB::PspoIIQ-yfp (kan), lacA::PgerE-yfp (tet)* | This work |
| **BAM719** | *ydiH::erm, yycR::PsspB-rbsopt-cfp (phleo), amyE::PspoIID-mCherry(spec), pelB::PspoIIQ-yfp (kan), lacA::PgerE-yfp (tet)* | This work |
| **BAM720** | *yerC::erm, yycR::PsspB-rbsopt-cfp (phleo), amyE::PspoIID-mCherry(spec), pelB::PspoIIQ-yfp (kan), lacA::PgerE-yfp (tet)* | This work |
| **BAM721** | *ygzB::erm, yycR::PsspB-rbsopt-cfp (phleo), amyE::PspoIID-mCherry(spec), pelB::PspoIIQ-yfp (kan), lacA::PgerE-yfp (tet)* | This work |
| **BAM722** | *ylbC::erm, yycR::PsspB-rbsopt-cfp (phleo), amyE::PspoIID-mCherry(spec), pelB::PspoIIQ-yfp (kan), lacA::PgerE-yfp (tet)* | This work |
| **BAM723** | *yqzE::erm, yycR::PsspB-rbsopt-cfp (phleo), amyE::PspoIID-mCherry(spec), pelB::PspoIIQ-yfp (kan), lacA::PgerE-yfp (tet)* | This work |
| **BAM724** | *nrnA::erm, yycR::PsspB-rbsopt-cfp (phleo), amyE::PspoIID-mCherry(spec), pelB::PspoIIQ-yfp (kan), lacA::PgerE-yfp (tet)* | This work |
| **BAM725** | *ytpI::erm, yycR::PsspB-rbsopt-cfp (phleo), amyE::PspoIID-mCherry(spec), pelB::PspoIIQ-yfp (kan), lacA::PgerE-yfp (tet)* | This work |
| **BAM726** | *ytxG::erm, yycR::PsspB-rbsopt-cfp (phleo), amyE::PspoIID-mCherry(spec), pelB::PspoIIQ-yfp (kan), lacA::PgerE-yfp (tet)* | This work |
| **BAM727** | *ywmB::erm, yycR::PsspB-rbsopt-cfp (phleo), amyE::PspoIID-mCherry(spec), pelB::PspoIIQ-yfp (kan), lacA::PgerE-yfp (tet)* | This work |
| **BAM728** | *speE::erm, yycR::PsspB-rbsopt-cfp (phleo), amyE::PspoIID-mCherry(spec), pelB::PspoIIQ-yfp (kan), lacA::PgerE-yfp (tet)* | This work |
| **BAM729** | *gidA::erm, yycR::PsspB-rbsopt-cfp (phleo), amyE::PspoIID-mCherry(spec), pelB::PspoIIQ-yfp (kan), lacA::PgerE-yfp (tet)* | This work |
| **BAM730** | *trmE::erm, yycR::PsspB-rbsopt-cfp (phleo), amyE::PspoIID-mCherry(spec), pelB::PspoIIQ-yfp (kan), lacA::PgerE-yfp (tet)* | This work |
| **BAM731** | *yqhT::erm, yycR::PsspB-rbsopt-cfp (phleo), amyE::PspoIID-mCherry(spec), pelB::PspoIIQ-yfp (kan), lacA::PgerE-yfp (tet)* | This work |
| **BAM732** | *resA::erm, yycR::PsspB-rbsopt-cfp (phleo), amyE::PspoIID-mCherry(spec), pelB::PspoIIQ-yfp (kan), lacA::PgerE-yfp (tet)* | This work |
| **BAM733** | *dacB::erm, yycR::PsspB-rbsopt-cfp (phleo), amyE::PspoIID-mCherry(spec), pelB::PspoIIQ-yfp (kan), lacA::PgerE-yfp (tet)* | This work |
| **BAM737** | *yaaD::erm, yycR::PsspB-rbsopt-cfp (phleo), amyE::PspoIID-mCherry(spec), pelB::PspoIIQ-yfp (kan), lacA::PgerE-yfp (tet)* | This work |
| **BAM738** | *defB::erm, yycR::PsspB-rbsopt-cfp (phleo), amyE::PspoIID-mCherry(spec), pelB::PspoIIQ-yfp (kan), lacA::PgerE-yfp (tet)* | This work |
| **BAM739** | *rasP::erm, yycR::PsspB-rbsopt-cfp (phleo), amyE::PspoIID-mCherry(spec), pelB::PspoIIQ-yfp (kan), lacA::PgerE-yfp (tet)* | This work |
| **BAM740** | *resC::erm,yycR::PsspB-rbsopt-cfp (phleo), amyE::PspoIID-mCherry(spec), pelB::PspoIIQ-yfp (kan), lacA::PgerE-yfp (tet)* | This work |
| **BAM741** | *resB::erm, yycR::PsspB-rbsopt-cfp (phleo), amyE::PspoIID-mCherry(spec), pelB::PspoIIQ-yfp (kan), lacA::PgerE-yfp (tet)* | This work |
| **BAM742** | *acsA::erm, yycR::PsspB-rbsopt-cfp (phleo), amyE::PspoIID-mCherry(spec), pelB::PspoIIQ-yfp (kan), lacA::PgerE-yfp (tet)* | This work |
| **BAM743** | *gtaB::erm, yycR::PsspB-rbsopt-cfp (phleo), amyE::PspoIID-mCherry(spec), pelB::PspoIIQ-yfp (kan), lacA::PgerE-yfp (tet)* | This work |
| **BAM744** | *iolR::erm, yycR::PsspB-rbsopt-cfp (phleo), amyE::PspoIID-mCherry(spec), pelB::PspoIIQ-yfp (kan), lacA::PgerE-yfp (tet)* | This work |
| **BAM745** | *mcsA::erm, yycR::PsspB-rbsopt-cfp (phleo), amyE::PspoIID-mCherry(spec), pelB::PspoIIQ-yfp (kan), lacA::PgerE-yfp (tet)* | This work |
| **BAM749** | *gapB::erm, yycR::PsspB-rbsopt-cfp (phleo), amyE::PspoIID-mCherry(spec), pelB::PspoIIQ-yfp (kan), lacA::PgerE-yfp (tet)* | This work |
| **BAM750** | *smpB::erm, yycR::PsspB-rbsopt-cfp (phleo), amyE::PspoIID-mCherry(spec), pelB::PspoIIQ-yfp (kan), lacA::PgerE-yfp (tet)* | This work |
| **BAM751** | *araR::erm, yycR::PsspB-rbsopt-cfp (phleo), amyE::PspoIID-mCherry(spec), pelB::PspoIIQ-yfp (kan), lacA::PgerE-yfp (tet)* | This work |
| **BAM752** | *minJ::erm, yycR::PsspB-rbsopt-cfp (phleo), amyE::PspoIID-mCherry(spec), pelB::PspoIIQ-yfp (kan), lacA::PgerE-yfp (tet)* | This work |
| **BAM822** | *clpC::erm, yycR::PsspB-rbsopt-cfp (phleo), amyE::PspoIID-mCherry(spec), pelB::PspoIIQ-yfp (kan), lacA::PgerE-yfp (tet)* | This work |
| **BAM823** | *asnO::erm, yycR::PsspB-rbsopt-cfp (phleo), amyE::PspoIID-mCherry(spec), pelB::PspoIIQ-yfp (kan), lacA::PgerE-yfp (tet)* | This work |
| **BAM825** | *uppP::erm, yycR::PsspB-rbsopt-cfp (phleo), amyE::PspoIID-mCherry(spec), pelB::PspoIIQ-yfp (kan), lacA::PgerE-yfp (tet)* | This work |
| **BAM826** | *ylbJ::erm, yycR::PsspB-rbsopt-cfp (phleo), amyE::PspoIID-mCherry(spec), pelB::PspoIIQ-yfp (kan), lacA::PgerE-yfp (tet)* | This work |
| **BAM827** | *menH::erm, yycR::PsspB-rbsopt-cfp (phleo), amyE::PspoIID-mCherry(spec), pelB::PspoIIQ-yfp (kan), lacA::PgerE-yfp (tet)* | This work |
| **BAM828** | *ytvI::erm, yycR::PsspB-rbsopt-cfp (phleo), amyE::PspoIID-mCherry(spec), pelB::PspoIIQ-yfp (kan), lacA::PgerE-yfp (tet)* | This work |
| **BAM830** | *ydcC::erm, yycR::PsspB-rbsopt-cfp (phleo), amyE::PspoIID-mCherry(spec), pelB::PspoIIQ-yfp (kan), lacA::PgerE-yfp (tet)* | This work |
| **BAM831** | *ycgM::erm, yycR::PsspB-rbsopt-cfp (phleo), amyE::PspoIID-mCherry(spec), pelB::PspoIIQ-yfp (kan), lacA::PgerE-yfp (tet)* | This work |
| **BAM832** | *yqfD::erm, yycR::PsspB-rbsopt-cfp (phleo), amyE::PspoIID-mCherry(spec), pelB::PspoIIQ-yfp (kan), lacA::PgerE-yfp (tet)* | This work |
| **BAM859** | *sacA::PgcaD-lacZ (phleo)* | This work |
| **BAM862** | Δ*spoIIT(63aa)::lox72* | This work |
| **BAM872** | *ycgO::Pspank-spoIIT (cat),* Δ*spoIIT(63aa)::lox72* | This work |
| **BAM873** | *spoIIR::erm-HI* | This work |
| **BAM875** | *sigE-gfp (spec)* | Fujita and Losick 2002 |
| **BAM876** | *sigE-gfp (spec), spoIIR::erm-HI* | This work |
| **BAM877** | *sigE-gfp (spec),* Δ*spoIIT(63aa)::lox72* | This work |
| eske et al. 2015erry (spec)] npublished) into the lacA::tet integration vector.lation by exhaustion. functions suggested by **BAM882** | *pelB::PspoIIQ-yfp (kan), amyE::PspoIID-mcherry (spec)* | Sullivan et al. 2009 |
| **BAM883** | *pelB::PspoIIQ-yfp (kan), amyE::PspoIID-mcherry (spec),* Δ*spoIIT(63aa)::lox72* | This work |
| **BAM884** | *pelB::PspoIIQ-yfp (kan), amyE::PspoIID-mcherry (spec), ycgO::Pspank-ywmB (cat),* Δ*spoIIT(63aa)::lox72* | This work |
| **BAM887** | *amyE::PspoIIT(small)-yfp (cat)* | This work |
| **BAM888** | *amyE::PspoIIT(large)-yfp (cat)* | This work |
| **BAM889** | *amyE::PspoIIT(small)-yfp (cat), sigF::erm-HI* | This work |
| **BAM890** | *amyE::PspoIIT(large)-yfp (cat), sigF::erm-HI* | This work |
| **BAM892** | *spoIIR-his6 (spec)* | This work |
| **BAM899** | *amyE::spoIID-lacZ (cat), pDG178(Pspac-spoIIGAB kan)* | Stragier et al. 1988 |
| **BAM900** | *amyE::spoIID-lacZ (cat), thrC::Pspac-spoIIR (erm), pDG178(Pspac-spoIIGAB kan)* | Stragier et al. 1988 |
| **BAM901** | *amyE::spoIID-lacZ (cat), thrC::Pspac-spoIIR (erm), pDG178(Pspac-spoIIGAB kan),* Δ*spoIIT(63aa)::lox72* | This work |
| **BAM902** | *yaaD::erm* | This work |
| **BAM906** | *rex::erm* | This work |
| **BAM907** | *yerC::erm* | This work |
| **BAM909** | *pgcA::erm* | This work |
| **BAM910** | *defB::erm* | This work |
| **BAM912** | *rasP::erm* | This work |
| **BAM916** | *dacB::erm* | This work |
| **BAM917** | *yqhT::erm* | This work |
| **BAM918** | *spoIIIL::erm* | This work |
| **BAM919** | *gapB::erm* | This work |
| **BAM920** | *nrnA::erm* | This work |
| **BAM922** | Δ*spoIIT(63aa)::lox72, spoIIR-his6 (spec)* | This work |
| **BAM927** | *ytxG::erm* | This work |
| **BAM928** | *smpB::erm* | This work |
| **BAM929** | *araR::erm* | This work |
| **BAM930** | *minJ::erm* | This work |
| **BAM931** | *gtaB::erm* | This work |
| **BAM932** | *spoIIT::erm* | This work |
| **BAM933** | *speE::erm* | This work |
| **BAM934** | *iolR::erm* | This work |
| **BAM935** | *gidA::erm* | This work |
| **BAM936** | *trmE::erm* | This work |
| **BAM938** | *menH::erm* | This work |
| **BAM939** | *clpC::erm* | This work |
| **BAM940** | *ecsB::erm* | This work |
| **BAM941** | *asnO::erm* | This work |
| **BAM942** | *skfF::erm* | This work |
| **BAM943** | *mntR::erm* | This work |
| **BAM944** | *lgt::erm* | This work |
| **BAM945** | *ymdB::erm* | This work |
| **BAM947** | *sigL::erm; pelB::PspoIIQ-YFP (kan)* | This work |
| **BAM949** | *sigL::erm* | This work |
| **BAM950** | *scoC::erm* | This work |
| **BAM951** | *prsW::erm* | This work |
| **BAM952** | *sigW::erm* | This work |
| **BAM953** | *sda::erm* | This work |
| **BAM954** | *ysaA::erm* | This work |
| **BAM955** | *bmrA::erm* | This work |
| **BAM956** | *pelB::PspoIIQ-CFP (kan); sacA::Pveg-mCherry (tet)* | eske et al. 2015erry (spec)] npublished) into the lacA::tet integration vector.lation by exhaustion. functions suggested by  Meeske et al. 2015 |
| **BAM957** | *pelB::PspoIIQ-YFP (kan)* | Sullivan et al. 2009 |
| **BAM958** | *uppP::erm* | This work |
| **BAM959** | *ylbJ::erm* | This work |
| **BAM960** | *ytvI::erm* | This work |
| **BAM961** | *dgkA::erm* | This work |
| **BAM962** | *yabQ::erm* | This work |
| **BAM963** | *ftsH::erm* | This work |
| **BAM964** | *ecsA::erm* | This work |
| **BAM965** | *efp::erm* | This work |
| **BAM966** | *ydcC::erm* | This work |
| **BAM968** | *cotE::erm* | This work |
| **BAM969** | *putB::erm* | This work |
| **BAM970** | *pdaB::erm* | This work |
| **BAM971** | *rsfA::erm* | This work |
| **BAM972** | *prpC::erm* | This work |
| **BAM973** | *ytrH::erm* | This work |
| **BAM974** | *yqfD::erm* | This work |
| **BAM975** | *gerM::erm* | This work |
| **BAM976** | *yabP::erm* | This work |
| **BAM977** | *kbaA::erm* | This work |
| **BAM978** | *skfE::erm* | This work |
| **BAM979** | *divIVA::erm* | This work |
| **BAM980** | *yqfC::erm* | This work |
| **BAM981** | *ytrI::erm* | This work |
| **BAM995** | *ywhA::erm* | This work |
| **BAM1007** | *sacA::PgcaD-lacZ (phleo), ykvU::erm* | This work |
| **BAM1008** | *sacA::PgcaD-lacZ (phleo), ykvI:erm* | This work |
| **BAM1009** | *sacA::PgcaD-lacZ (phleo), yrbG::erm* | This work |
| **BAM1010** | *sacA::PgcaD-lacZ (phleo), yyaC::erm* | This work |
| **BAM1011** | *sacA::PgcaD-lacZ (phleo), ykoN::erm* | This work |
| **BAM1012** | *sacA::PgcaD-lacZ (phleo), prpB::erm* | This work |
| **BAM1013** | *sacA::PgcaD-lacZ (phleo), yqhH::erm* | This work |
| **BAM1014** | *sacA::PgcaD-lacZ (phleo), yqgE::erm* | This work |
| **BAM1017** | *ykvU::erm, yycR::PsspB-rbsopt-cfp (phleo), amyE::PspoIID-mCherry(spec), pelB::PspoIIQ-yfp (kan), lacA::PgerE-yfp (tet)* | This work |
| **BAM1018** | *ykvI::erm, yycR::PsspB-rbsopt-cfp (phleo), amyE::PspoIID-mCherry(spec), pelB::PspoIIQ-yfp (kan), lacA::PgerE-yfp (tet)* | This work |
| **BAM1019** | *yrbG::erm, yycR::PsspB-rbsopt-cfp (phleo), amyE::PspoIID-mCherry(spec), pelB::PspoIIQ-yfp (kan), lacA::PgerE-yfp (tet)* | This work |
| **BAM1020** | *yyaC::erm, yycR::PsspB-rbsopt-cfp (phleo), amyE::PspoIID-mCherry(spec), pelB::PspoIIQ-yfp (kan), lacA::PgerE-yfp (tet)* | This work |
| **BAM1025** | *kinA Ω Phyperspank-kinA (spec)* | Fujita and Losick 2005 |
| **BAM1026** | *amyE::Phyperspank-yyaJ (spec)* | This work |
| **BAM1027** | *amyE::Phyperspank-slrA (spec)* | This work |
| **BAM1031** | *spoIIR::erm* | This work |
| **BAM1032** | *pelB::PspoIIQ-yfp (kan), amyE::PspoIID-mcherry (spec), spoIIR::erm* | This work |
| **BAM1041** | *amyE::Phyperspank-optRBS-yhzC (spec)* | This work |
| **BAM1043** | *amyE::Phyperspank-optRBS-nhaC (spec)* | This work |
| **BAM1052** | *magellan6x-dtpT (spec)* | This work |
| **BAM1053** | *magellan6x-helD (spec)* | This work |
| **BAM1047** | *amyE::Phyperspank-optRBS-yhzC (spec), pelB::PspoIIQ-yfp (kan)* | This work |
| **BAM1057** | *amyE::Phyperspank-optRBS-nhaC, pelB::PspoIIQ-yfp (kan)* | This work |
| **BAM1058** | *magellan6x-helD (spec), pelB::PspoIIQ-yfp (kan)* | This work |
| **BAM692** | Δ*yqzE* | This work |
| **BAM694** | Δ*yqhT* | This work |
| **BAM700** | Δ*rex (ydiH)* | This work |
| **BAM701** | Δ*ytxG* | This work |
| **BAM702** | Δ*speE* | This work |
| **BAM703** | Δ*gidA* | This work |
| **BAM704** | Δ*trmE* | This work |
| **ΒΑΜ705** | Δ*resA* | This work |
| **BAM709** | Δ*rasP* | This work |
| **BAM712** | Δ*gapB* | This work |
| **BAM714** | Δ*smpB* | This work |
| **BAM1088** | Δ*yerC* | This work |
| **BAM1089** | Δ*pgcA* | This work |
| **BAM1090** | Δ*defB* | This work |
| **BAM1091** | Δ*nrnA* | This work |
| **BAM1092** | Δ*araR* | This work |
| **BAM1093** | Δ*minJ* | This work |
| **BAM1094** | Δ*gtaB* | This work |
| **BAM1095** | Δ*iolR* | This work |
| **BAM1097** | Δ*ubiE (menH)* | This work |
| **BAM1098** | Δ*miaA* | This work |
| **BAM1099** | Δ*ndk* | This work |

* In PY79 background
