# Supplementary material for: High-Throughput Genetic Screens Identify a Large and Diverse Collection of New Sporulation Genes in Bacillus subtilis
Source: PLoS Biol. 2016 Jan 6;14(1):e1002341. doi: 10.1371/journal.pbio.1002341 (PMC4703394; doi:10.1371/journal.pbio.1002341)
Supplement: S5 Table — (DOCX) [file pbio.1002341.s018.docx]

Table S6. List of plasmids used in this study.

| **Plasmid name** | **Description** |
| --- | --- |
| pAM139 | lacA::PgerE-yfp (tet) |
| pCR100 | amyE::PspoIID-mcherry (spec) |
| pER77 | sacA::PgcaD-lacZ (phleo) |
| pCR218 | yhdG::PspoIIIL-spoIIIL (tet) |
| pCR219 | yhdG::Pspank-optRBS-spoIIIL (lacI) (phleo) |
| pCR220 | amyE::PspoIIIL-optRBS-yfp (spec) |
| pAM161 | amyE::Phyperspank-yyaJ (spec) |
| pAM162 | amyE::Phyperspank-slrA (spec) |
| pAM166 | ycgO::Pspank-spoIIT (cat) |
| pAM175 | amyE::PspoIIT(small)-yfp (cat) |
| pAM176 | amyE::PspoIIT(large)-yfp (cat) |
| pAM193 | amyE::Phyperspank-yhzC (spec) |
| pAM195 | amyE::Phyperspank-nhaC (spec) |
|  |  |
|  |  |
|  |  |
|  |  |
|  |  |
|  |  |
|  |  |
