# Supplementary material for: High-Throughput Genetic Screens Identify a Large and Diverse Collection of New Sporulation Genes in Bacillus subtilis
Source: PLoS Biol. 2016 Jan 6;14(1):e1002341. doi: 10.1371/journal.pbio.1002341 (PMC4703394; doi:10.1371/journal.pbio.1002341)
Supplement: S6 Table — (DOCX) [file pbio.1002341.s019.docx]

Table S7. List of oligonucleotide primers used in this study.

| **Primer name** | **Sequence*** |
| --- | --- |
| oCR467 | cgcGAATTCatcaacttaagagtgtcaacgg |
| oCR475 | cgcGGATCCgtcctccattttttgaaggacc |
| oCR476 | cgcAAGCTTacataaggaggaactactatgaaaacgaatgattatgttaaatatatg |
| oCR477 | cgcGCTAGCgtcctccattttttgaaggacc |
| oCR482 | cgcAAGCTTatcattcaattattatgagtgttgtc |
| oCR483 | cgcAAGCTTacataaggaggaactactatgagtaaaggagaagaacttttc |
| oDR078 | gccGGATCCttatttgtatagttcatccatgcc |
| oAM428 | gcgAAGCTTacataaggaggaactactatgaatacgatcttcaaacaaaag |
| oAM429 | cgcGCTAGCttaaatctgttttgcattcgtatt |
| oAM430 | gcgAAGCTTacataaggaggaactactatgaaaactcatgttaaaaaagatttg |
| oAM431 | cgcGCTAGCctagtcttgccggacggt |
| oAM437 | gcgCCCGGGacataaggaggaactactatgaagaaaaaacaagtaagccac |
| oAM438 | cgcGCTAGCttaatattcagtcgtaacgattgg |
| oAM459 | cgcGAATTCatgagtgtattgggacagc |
| oAM460 | cgcAAGCTTgttcatctctcccccttatc |
| oAM461 | ctcGAATTCttcaataggaag |
| oAM433 | gaagaagttgttgagaaagc |
| oAM434 | tcctgcctttcctccctccatgttcatctctccccctt |
| oAM435 | gagagcacagatacggcgaaagaattttatcaaaaagtgcaacg |
| oAM436 | ttgtgactgcatatcagtcg |
| oAM468 | gcaaaggggatgtaaacatg |
| oAM469 | tactgagcgagggagcagaattagtgatggtgatggtgatgggaaaaaaggtcggaaatcc |
| oAM470 | tagttgaccagtgctccctgtcatctggtctaaaaatagatttg |
| oAM471 | gcagctcttcacataatcag |
| oAM489 | ttgtgagcggataacaattaacataaggaggaactactatgaaagagaaaaaatcgtacac |
| oAM490 | ccgaattagcttgcatgcggttattcgctctgcttgagc |
| oAM493 | ttgtgagcggataacaattaacataaggaggaactactatggattctcaaaaaaagctg |
| oAM494 | ccgaattagcttgcatgcggttattttttaacaaatccaatcaaaatgg |
| oAM497 | gacacatagatggcgtcgct |
| oAM498 | aacaaagaaaaacacatttttttgtc |
| oAM505 | caatcaccgcgttcccttg |
| oAM506 | agcgacgccatctatgtgtcataatacttgtgtcaatataatttatac |
| oAM507 | caaaaaaatgtgtttttctttgttataagaatatgatttttataatacactttt |
| oAM508 | tcatcgcgcgtttgatcac |
| oAM509 | ggaaacattcgcttctcaag |
| oAM510 | agcgacgccatctatgtgtcatattaaatatatgatgcaaactgg |
| oAM511 | caaaaaaatgtgtttttctttgttatgtatccaattatattttacagta |
| oAM512 | gtaacatcaagaaatgcaagg |
| oDR1049 | gagggaggaaaggcagga |
| oDR1050 | cgccgtatctgtgctctc |
| oJM28 | ttctgctccctcgctcag |
| oJM29 | cagggagcactggtcaac |

*Capital letters indicate restriction endonuclease cleavage sites
